# Functional Polymers for Ionic Thermoelectrics: Multiscale Design Strategies for Ion Dynamics, Mechanics, and Energy Harvesting

**DOI:** 10.1002/adma.202519451

**Published:** 2026-02-06

**Authors:** Sungryong Kim, Jin Han Kwon, Juyoung Kang, Hong Chul Moon

**Affiliations:** ^1^ Department of Chemical & Biomolecular Engineering Korea Advanced Institute of Science and Technology Daejeon Republic of Korea

**Keywords:** energy harvesting, functional polymer, ionic thermoelectrics, multi‐scale engineering, quasi‐solid‐state ionic conductor, thermodiffusion effect, thermogalvanic effect

## Abstract

The efficient conversion of dissipated heat into useful electrical energy has emerged as a promising approach for sustainable energy technologies. Ionic thermoelectrics (iTEs) are particularly attractive because they generate substantial thermo‐voltages, effectively harvest low‐grade heat, and offer advantages such as cost‐effectiveness, easy scalability, and remarkable performance. Unlike liquid‐state platforms, quasi‐solid‐state iTEs exhibit properties that are critically governed by the polymer matrix and polymer‐ion interactions, which are closely related to the overall device performance. Consequently, the use of functional polymers effectively improves the characteristics and performance of quasi‐solid‐state iTEs. Therefore, this paper highlights the impact of the polymer matrix on performance and mechanical properties in iTEs from molecular‐, micro‐, to macro‐scale engineering. Particular emphasis is placed on clarifying the role of polymers at various scales to provide an in‐depth understanding of performance enhancement. Furthermore, the current challenges and prospective research directions are discussed, offering guidance toward the development of next‐generation iTE‐based energy harvesting platforms.

## Graphical abstract

This review highlights the impact of the polymers at the molecular‐, micro‐, and macro‐scale engineering. Particular emphasis is placed on their roles at different scales to provide an in‐depth understanding of performance improvement. In addition, current challenges and potential research approaches are discussed, outlining directions for the future development of iTE‐based energy harvesting systems.

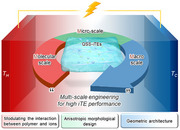

**Abbreviations**
*α*
: thermopower
*η*
: thermal conversion efficiency
*η_r_*
: carnot relative efficiency
*κ*
: thermal conductivity
*σ_i_*
: ionic conductivity
AETA: 2‐(acryloyloxy)ethyl]trimethylammonium chloride
ANF: deprotonated aramid nanofibers
BF_4_
: tetrafluoroborate
BQ: *p*‐Benzoquinone
ChCl: choline chloride
DCA: dicyanamide
DMAEA‐Q: methyl chloride quaternized *N*, *N*‐dimethylamino ethylacrylate
EDL: electric double layer
EDLC: electric double‐layer capacitor
EMIM: 1‐ethyl‐3‐methylimidazolium
EMIMOTF: trifluoromethanesulfonate
eTEs: electronic thermoelectrics
hPEI: hyperbranced poly (ethyleneimine)
HQ: *p*‐Hydroquinone
IPN: interpenetrating network
iTEs: ionic thermoelectrics
LS: Iiquid‐state
MC: methyl cellulose
MIEC: mixed ionic‐electronic conductor
P(([APTA][TFSI])‐*co*‐MA): Poly (methyl acrylate‐*co*‐5‐(acryloyloxy)pentyl)‐trimethylammonium bis(trifluoromethane) sul‐fonimide)
P(([EMIM][SPA])‐*co*‐MA): Poly (methyl acrylate‐*co*‐3‐sulfopropyl acrylate 1‐ethyl‐3‐methyl imidazolium)
P(AA‐*co*‐HEA): Poly (acrylic acid‐*co*‐hydroxyethyl acrylate)
P(AAm‐*co*‐AMPS): Poly (2‐acrylamide‐2‐methylpropane sulfonic acid‐*co*‐acrylamide)
P(NIPAM‐*co*‐AA): Poly(*N*‐isopropylacrylamide‐*co*‐acrylic acid)
PA: phytic acid
PAA: Poly (acrylic acid)
PAA‐*co*‐AAm: Poly (acrylic acid‐*co*‐acrylamide)
PAAm: Poly (acrylamide)
PAAm‐*co*‐AAK: Poly (acrylamide‐*co*‐potassium acrylate)
PAMPS: Poly (2‐acrylamido‐2‐methyl‐1‐propanesulfonic acid)
PANI: Poly (aniline)
PDAC: Poly ([2‐(Acryloyloxy)ethyl]dimethylammonium chloride)
PDMAPS: Poly ([2‐(methacryloyloxy)ethyl]dimethyl‐(3‐sulfopropyl)ammonium hydroxide)
PDMS: Poly (dimethylsiloxane)
PEDOT:PSS: Poly (2,3‐dihydrothieno‐1,4‐dioxin)‐Poly(styrenesulfonate)
PEG: Poly (ethylene glycol)
*PF*
: power factor
PLAM: Poly (2‐acrylamide‐2‐methyl‐1‐propane sulfonic acid‐*co*‐3‐sulfopropyl methacrylate lithium)
PMACP: Poly (2‐methacryloyloxyethyl phosphorylcholine)
PNAGA: Poly (N‐acryloyl glycinamid)
PNAGA‐F68: Poly (N‐acryloyl glycinamide‐*co*‐diacrylate capped Pluronic F68)
PNIPAM: Poly (*N*‐isopropylacrylamide)
PSBMA: Poly [2‐(methacryloyloxy)‐ethyl]dimethyl‐(3‐sulfo‐propyl) ammonium hydroxide]
PSS‐Na: Poly (sodium 4‐styrenesulfonate)
PVA: Poly (vinyl alcohol)
PVDF‐HFP: Poly (vinylidene fluoride‐*co*‐hexafluoropropylene)
QSS: quasi‐solid‐state
RH: relative humidity
TD: thermodiffusion
TFSI: Bis(trifluoromethanesulfonyl)imide
TG: thermogalvanic
TOBC: 2,2,6,6‐Tetramethylpiperidine‐1‐oxyl radical‐oxidized carboxylated bacterial cellulose
TPFPB: Tris(pentafluorophenyl)borane
VDT: vinyl diaminotriazine
WPU: water‐borne Poly (urethane)
*ZT*
: figure of merit

## 1 Introduction

Industrial growth requires substantial energy; however, conventional fossil fuel‐based sources are becoming progressively exhausted. As an alternative, increasing attention has been directed toward renewable energy technologies [1, 2, 3, 4, 5, 6, 7, 8, 9]. Among them, thermal energy is particularly attractive because it is an abundant renewable source readily available in our surroundings, for example, low‐grade heat (e.g., body or geothermal sources) [1, 2, 3] and high‐grade heat (e.g., industrial waste) [4, 5]. Over the past decades, researchers have attempted to harness waste heat by converting it into usable electrical energy via thermoelectric (TE) effects [6, 7, 8, 9].

**FIGURE 1:**
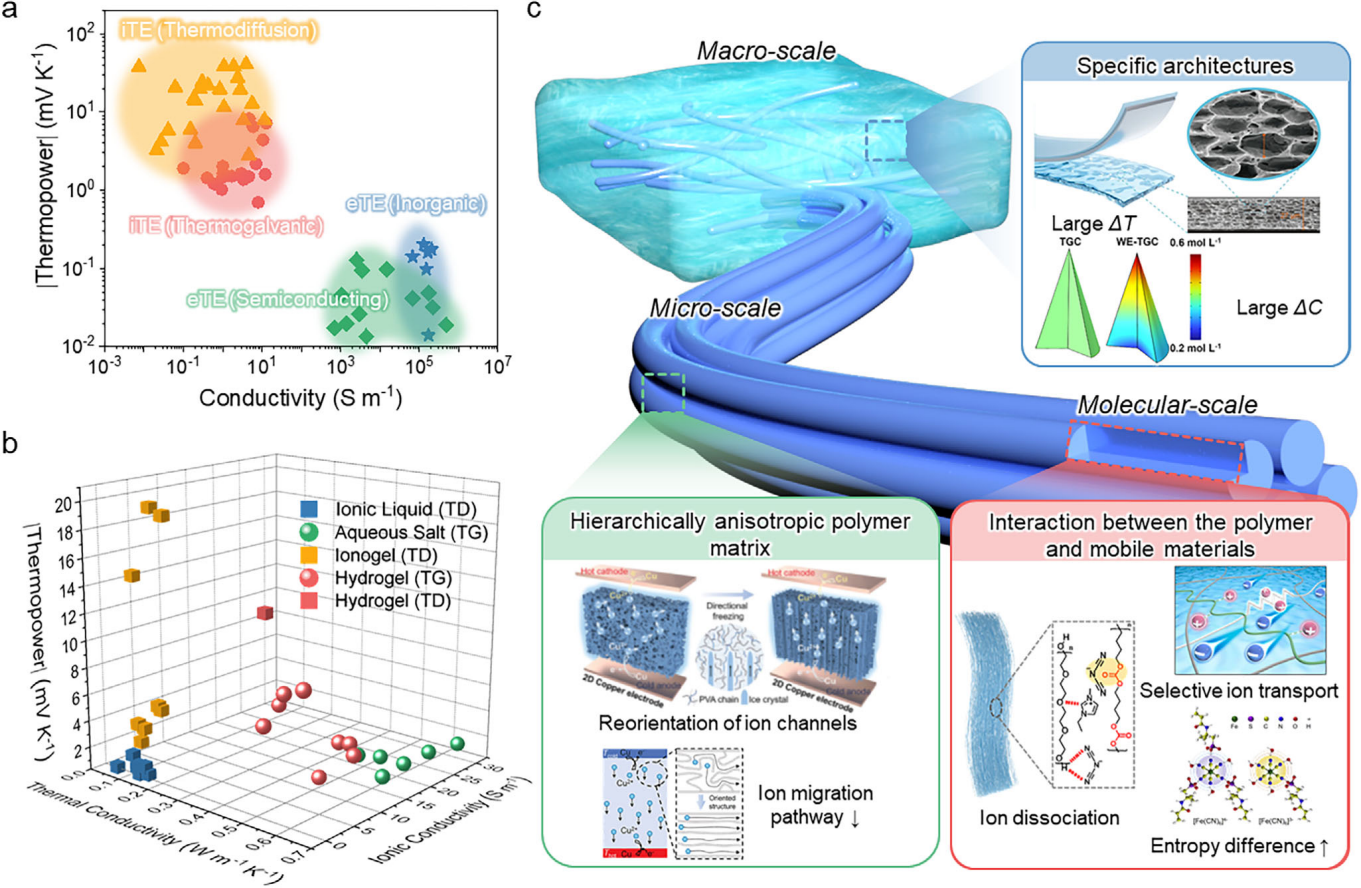
(a) *α* and *σ_i_* of reported iTEs and eTEs [15, 16, 17, 18, 19, 20, 21, 22, 23, 24, 25, 26, 27, 28, 29, 30, 31, 32, 38, 39, 40, 41, 42, 43, 44, 45, 46, 47, 48, 49, 50, 51, 52, 53, 54, 55, 56, 57, 58, 59, 60, 61, 62, 63, 64, 65, 66, 67, 68, 69, 70, 71, 72, 73, 74, 75, 76, 77, 78, 79, 80, 81, 82, 83, 84, 85, 86, 87, 88, 89, 90, 91, 92, 93]. (b) Comparison of *α*, *σ_i_*, and thermal conductivity in LS‐ionic conductors (aqueous salt and ionic liquid) and QSS‐ionic conductors (hydrogel and ionogel) [38, 41, 44, 49, 50, 56, 57, 58, 62, 72, 73, 147, 161, 162, 163, 165, 183, 197, 198, 199, 200]. (c) Schematic diagram of various strategies for improvement from molecular‐, micro, to macro‐scale engineering. Reproduced with permission [168]. Copyright 2025, Springer. Reproduced with permission [170]. Copyright 2025, Wiley‐VCH. Reproduced with permission [75]. Copyright 2024, Wiley‐VCH. Reproduced with permission [169]. Copyright 2025, American Chemical Society. Reproduced with permission [47]. Copyright 2022, Elsevier. Reproduced with permission [64]. Copyright 2021, Elsevier.

TE systems are generally classified into electronic TEs (eTEs) [7, 8] and ionic TEs (iTEs) [9, 10, 11, 12] depending on the type of charge carriers. In eTEs, electrons (holes) serve as the main charge carriers and migrate from the hot side to the cold side within the TE materials. Eventually, the electrons (holes) are transferred to the cold side electrode, which generates a potential difference between the electrodes by the Seebeck effect [13, 14]. However, eTEs typically produce only a few hundred µV K^−1^ thermo‐voltage (Figure 1a), reducing their practicality [15, 16, 17, 18, 19, 20, 21, 22, 23, 24, 25, 26, 27, 28, 29, 30, 31, 32]. To overcome this limitation and achieve a higher thermo‐voltage level suitable for practical uses, eTEs require multiple thermocouples (p‐ and n‐type integration) and a complex device structure/fabrication process [33, 34].

In contrast, iTEs rely on ions as the main charge carriers, which migrate via thermophoresis under a temperature gradient [35, 36, 37]. The utilization of ions produces significantly higher thermo‐voltage outputs, exceeding a few mV K^−1^ (Figure 1a) [38, 39, 40, 41, 42, 43, 44, 45, 46, 47, 48, 49, 50, 51, 52, 53, 54, 55, 56, 57, 58, 59, 60, 61, 62, 63, 64, 65, 66, 67, 68, 69, 70, 71, 72, 73, 74, 75, 76, 77, 78, 79, 80, 81, 82, 83, 84, 85, 86, 87, 88, 89, 90, 91, 92, 93]. This enhanced thermo‐voltage significantly decreases the number of thermocouples and simplifies the device fabrication process [94, 95]. Owing to their low cost and simple scalability, iTEs possess considerable potential for integration into practical energy harvesting modules [96, 97]. Moreover, their ability to convert even low‐grade thermal energy into electricity renders them particularly promising for wearable applications that exploit body heat [98, 99, 100].

According to their operating principles, iTEs function through either the thermodiffusion (TD) effect or the thermogalvanic (TG) effect. In the TD effect, mobile ions transport from the hot to the cold side and form an electric double layer (EDL) at the electrode interface. This establishes a potential difference between the electrodes without direct electron transfer from/to the electrodes [44, 45, 53, 57, 59, 101]. The working principle of the TD effect is quite similar to that of EDL capacitors (EDLCs) [102, 103]. On the other hand, the TG effect relies on temperature‐dependent reversible redox reactions at the electrodes, where redox couples mediate electron transfer to generate a potential difference [64, 65, 66, 67, 104]. These distinct mechanisms demand tailored strategies to enhance iTE performance.

From a materials perspective, two major categories of iTEs are liquid‐state (LS) and quasi‐solid‐state (QSS) systems. Typically, LS‐iTEs consist of salt‐containing aqueous/organic solutions, or ionic liquids, which serve as both the medium and charge carrier. Ion migration is generally facilitated in the LS, leading to high ionic conductivity (*σ_i_*) (Figure 1b) [38, 41, 44, 49, 50, 56, 57, 58, 62, 72, 73, 147, 161, 162, 163, 165, 183, 197, 198, 199, 200]. In contrast, QSS‐iTEs are fabricated by incorporating a polymer matrix into an LS‐ionic conductor. The introduction of a polymer matrix offers several advantages. For example, polymer‐ion interactions promote ion dissociation and enhance the diffusivity difference between cations and anions by weakening their electrostatic attraction at the molecular level. Moreover, micro‐ and macro‐scale engineering within the polymer matrix enables regulation of ionic and thermal diffusion, which is effective for controlling both the magnitude and sign of the thermo‐voltage [49, 51]. In addition, the mechanical robustness, stretchability, and toughness of the QSS‐iTEs expand their practical applicability, particularly in wearable devices [105, 106].

In recent decades, researchers have sought to improve the iTEs through molecular engineering [50, 56, 57, 58], electrode modification [107, 108], and hybrid integration with complementary energy harvesting systems (e.g., photocatalysts, moisture generators, and piezoelectric generators) [72, 109, 110, 111]. In accordance with these advancements, viewpoints improving iTEs from a device‐specific perspective have been reported. However, the polymer matrix, which provides the medium for ion migration, plays a crucial role in determining ionic transport, TE performance, and mechanical properties. Therefore, it is necessary to understand the performance enhancements of iTEs from the perspective of the polymer matrix.

Herein, this review systematically examines the pivotal role of polymer matrices in iTEs, with emphasis on their contribution to TE, electrical, and mechanical properties. Multi‐scale strategies, ranging from molecular‐ and micro‐scale engineering to macro‐scale architecture design, are discussed (Figure 1c). The impact of polymer‐ion interactions, anisotropic structures, and device‐level engineering is analyzed to provide in‐depth insights into performance optimization. Finally, we present the current challenges of iTE research and highlight future directions in multi‐scale polymer engineering, aiming to accelerate the realization of iTEs for next‐generation wearable technologies and sustainable energy applications.

## 2 Basic Principle of Ionic Thermoelectrics

### 2.1 Soret Effect and Coupled Thermal‐Ionic Transport

Mobile materials randomly diffuse within the medium (solvent and polymer matrix) via Brownian motion when the temperature between the ends of the medium is identical [101, 112]. However, in the 19th century, Ludwig and Soret discovered that when a temperature gradient arises between two ends of a medium, the chemical potential (*µ_i_*) of mobile materials becomes spatially asymmetric, leading to thermodiffusion [113, 114]. They expressed the relation between the concentration gradient (Δ*c_i_*) and the temperature gradient (Δ*T*) using Equation (1):

(1) $$\nabla \;c_{i}=-c_{i}S_{T,i}\nabla T$$

where *c_i_* is an initial concentration of species *i* under Δ*T* = 0, and *S_T,i_* represents the Soret coefficient of species *i*. The sign of *S_T,i_* indicates the direction of thermodiffusion, implying positive (negative) for accumulation of species *i* on the cold (hot) side [113, 114].

To explore this behavior, the *µ_i_* (*c_i_*, *T*) is expanded regarding *c_i_* and *T*. By combining this expansion with the Onsager linear relation ($J_{i}=-L_{i}\nabla \left(\frac{\mu_{i}}{T}\right))$ and the Gibbs‐Duhem relations for an ideal solution, the mass flux of species *i* is expressed as Equation (2) [115, 116]:

(2) $$J_{i}=-L_{i}\nabla \;\left(\frac{\mu_{i}}{T}\right)=-D_{i}\nabla c_{i}-c_{i}D_{i}S_{T,i}\nabla T$$

where *L_i_*, *D_i_* (= *L_i_k_B_*/*c_i_*), and *k_B_* are the Onsager linear transport coefficient, diffusivity of species *i*, and Boltzmann constant. This phenomenon (Soret effect) was described by Eastman in 1926 from a thermodynamic perspective, incorporating the concept “heat of transport” ($Q_{i}^{*}$) by Equation (3) [115]:

(3) $$S_{T,i}=\frac{Q_{i}^{*}}{k_{B}T^{2}}\;$$

Thus, $Q_{i}^{*}$ reflects how the enthalpy of a species changes along a temperature gradient. A larger positive $Q_{i}^{*}$ indicates that the species becomes energetically less stable at higher temperatures; hence, it preferentially migrates toward the colder region [115].

In contrast to neutral species, ions form an electric field in the medium due to the different mobilities and thermophoretic behaviors of cations and anions. To precisely reflect the behavior of ions under the effect of an electric field, an electrochemical potential (${\tilde{\mu }}_{i}=\mu_{i}+z_{i}e\phi$) is incorporated into the flux relation 4 [114, 115]:

(4) $$J_{i}=-L_{i}\nabla \;\left(\frac{{\tilde{\mu }}_{i}}{T}\right)=-D_{i}\nabla c_{i}-c_{i}D_{i}S_{T,i}\nabla T-z_{i}ev_{i}c_{i}\nabla \phi$$

where *z_i_*, *v_i_*, *e*, and ϕ are valence charge and mobility of species *i*, elementary charge, and electrostatic potential, respectively. In Equation (4), *J_i_* describes the transport of mobile materials with respect to diffusion (Fick's law) in the first term, thermophoresis in the second term, and drift under an electric field in the third term [115].

In open‐circuit conditions, the net charge flow becomes zero (Σ*z_i_J_i_* = 0). In bulk LS‐ionic conductors, ∇*c* is negligible [116]. Moreover, ions are generally composed of cation and anion pairs (1:1). Thus, if *z_cation_* is +1 and *z_anion_* is −1, the flux relation of all ions in the medium is expressed as follows 5 and 6:

(5) $$J_{+}-\;J_{-}=0$$

(6) $$e\left(c_{+}v_{+}+c_{-}v_{-}\right)\nabla \phi =\left(c_{+}D_{-}S_{T,\;-}-c_{-}D_{+}S_{T,+}\right)\nabla T$$

Equation (6) explicitly exhibits how the internal electric field arises from the difference in thermophoretic responses between the cations and anions. The thermopower (*α*) is defined as the ratio of the induced electrostatic potential gradient to the applied temperature gradient under open‐circuit conditions using the Nernst‐Einstein relation (*D_i_* = *v_i_k_B_T*) in Equation (7) [116]:

(7) $$\alpha =-\;\frac{\nabla \phi }{\nabla T}=\frac{\left(c_{+}D_{+}S_{T,+}-c_{-}D_{-}S_{T,-}\right)}{e\left(c_{+}v_{+}+c_{-}v_{-}\right)}=\frac{k_{B}T}{e}\;\frac{c_{+}v_{+}S_{T,+}-c_{-}v_{-}S_{T,-}}{c_{+}v_{+}+c_{-}v_{-}}$$

Each ion exhibits distinct mobility under an electric field. Therefore, when considering the Hittorf transport number (*t_±_* = *c_±_v_±_*/(*c_+_v_+_* +*c_–_v_–_*)), it can be expressed as follows, Equation (8) [116]:

(8) $$\frac{\Delta \phi }{\Delta T}=\frac{t_{+}Q_{+}^{*}-t_{-}Q_{-}^{*}}{eT}\;$$

Therefore, the mobility difference between cations and anions or ion‐medium interactions affects thermal voltage.

In addition, mobile ions have stronger electrostatic interactions in the QSS‐ionic conductors than in the LS‐ionic conductors due to reduced solvation by the interaction with polymers. Thus, they exhibit different behaviors compared to those of the LS‐ionic conductors [117, 118]. In 2021, Würger proposed a model of ion transport in polymers [119]. Mobile ions are localized in certain sites and jump to adjacent sites via hopping after activation. In the absence of a temperature gradient, forward and backward hopping occur at the same rate. As a result, ion hopping occurs randomly. However, when a temperature gradient is applied, the activation energy decreases at high‐temperature sites. Consequently, the forward hopping rate exceeds the backward hopping rate, driving ion transport to the cold side. Accordingly, Q* can be expressed as follows, Equation (9):

(9) $$Q^{*}=\Delta H+k_{B}T-\frac{d\Delta G}{dc}T\frac{dc}{dT}$$

where Δ*H* and Δ*G* denote the activation enthalpy and the free enthalpy of activation, respectively [120].

### 2.2 Working Mechanism of the Thermodiffusion Effect: EDL

**FIGURE 2:**
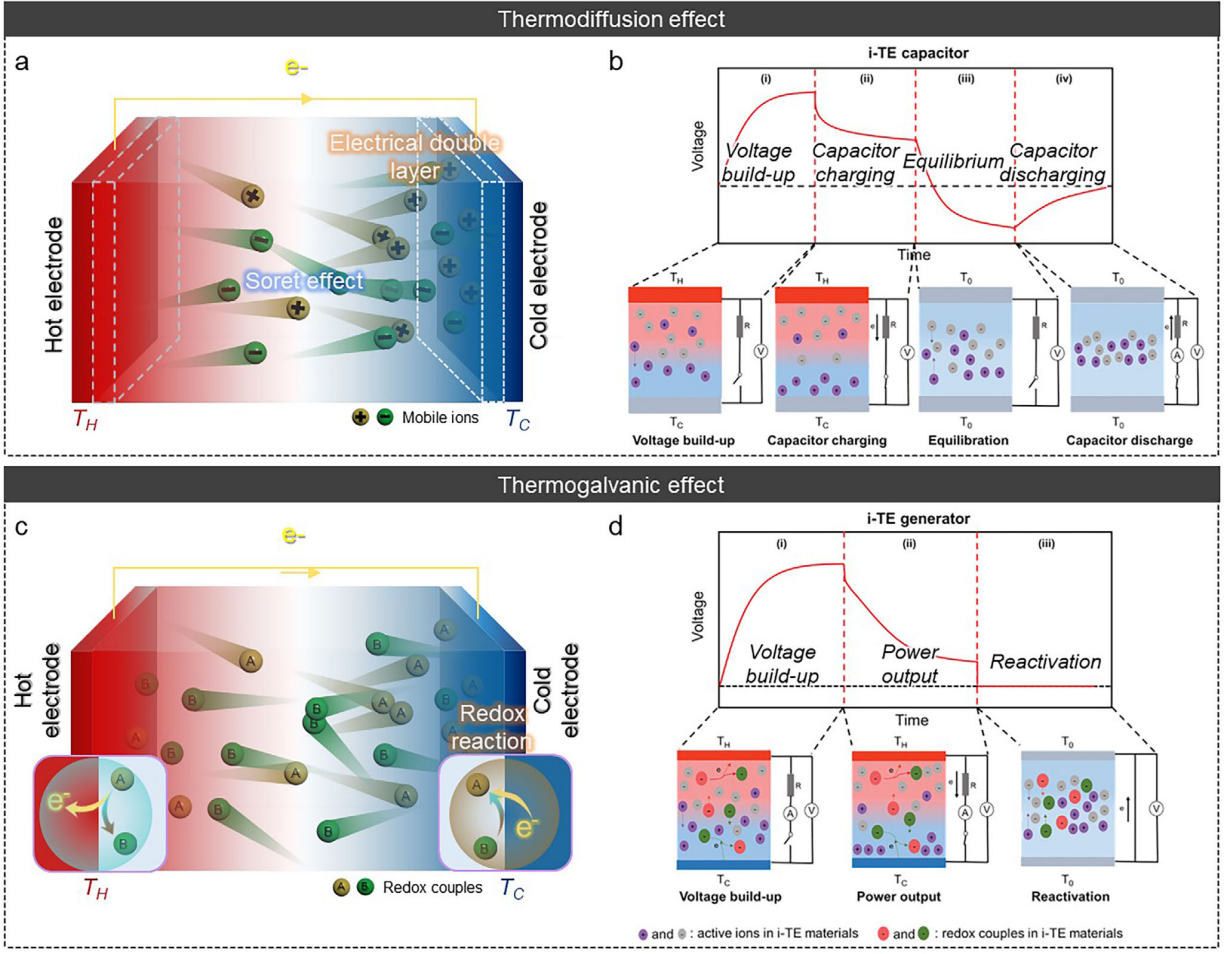
(a) and (c) Illustration of TD and TG effect. (b) and (d) Working principle and voltage profile of TD, TG, and synergistic effect. Reproduced with permission [101]. Copyright 2022, Wiley‐VCH.

The working step of a TD effect is illustrated in Figure 2a,b. 1) Mobile ions (both cations and anions) are transported to the cold side under a temperature gradient via thermophoresis [68, 101]. This transport results in the accumulation of ions at the interface of the cold side electrode. The accumulated ions establish an EDL with the electrode, leading to a potential difference between the hot and cold sides. Therefore, the “p‐ and n‐type” TE properties are defined by the charge of the dominant ion in the EDL (Table 1) [37, 39, 40, 41, 42, 43, 44, 45, 46, 47, 48, 49, 50, 51, 52, 53, 54, 55, 56, 57, 58, 96, 157]. As the ions that accumulate at the interface become saturated, the potential difference gradually stabilizes. Subsequently, 2) when an external circuit is connected to both electrodes, electrons flow from one side of the electrode to the other through the external circuit to compensate for the potential difference of the QSS‐ionic conductors, generating a potential difference at both electrodes. In this process, maximum power and energy are determined when the external load resistance is close to the Thevenin resistance of QSS‐ionic conductors [53, 121, 122]. After electron flow is complete, the external circuit is disconnected. Subsequently, 3) as the temperature gradient decreases, the ions that accumulate at the EDL are distributed in random directions and return to their initial states. The electrical potentials then decrease in the reverse direction because the electrons flowing to compensate for the potential difference within the QSS‐ionic conductors remain unable to flow. 4) When the external circuit is re‐connected to both electrodes to compensate for the potential difference within the QSS‐ionic conductors, the electrons flow in the opposite direction again, restoring the system to its initial state before the temperature gradient is applied.

**TABLE 1:** Comparison of TD effect (*α*, *σ_i_*, thermal conductivity (*κ*), power factor (*PF*), power density, and figure of merit (*ZT*)) of reported TD cells based on polymers.

Polymer	Ion species	Type	*α* (mV K^−1^)	*σ_i_* (S m^−1^)	*κ* (W m^−1^ K^−1^)	*PF* (mW m^−1^K^−2^)	Power density (W m^−2^)	*ZT*	Refs.
Poloxamer 407	LiCl	p	15.4	0.4	0.50	—	—	0.7	[37]
PANI/PAMPS	PA	p	8.1	0.2	0.45	1.6	—	1.0	[39]
P(AA‐*co*‐AAm)	H_2_SO_4_	p	40.6	3.9	0.46	6.5	0.006	4.0	[40]
Carboxyl‐Functionalized Bacterial Cellulose	EMIMDCA	p	11.5	7.9	0.23	0.5	—	—	[41]
Polyquaternium‐10	NaOH	p	24.2	0.3	—	—	—	—	[42]
PEDOT:PSS/PAMPS	LiTFSI	p	7.9	3.3	0.49	0.2	3.5	0.2	[43]
WPU	ChCl	p	19.5	0.8	0.20	—	—	0.5	[44]
Gelatin	EMIMDCA	p	22.1	0.4	—	—	0.01	—	[45]
PAA/PEG	NaCl	p	3.3	0.05	0.32	0.0002	—	0.2	[46]
PVA	HCl	p	38.2	1.9	0.46	—	—	—	[51]
PVDF‐HFP	NaTFSI	p	20.0	0.3	0.17	—	—	0.2	[49]
Gelatin/PAAm	LiCl/Li_2_SO_4_	p	10.4	2.4	0.53	0.1	0.09	—	[52]
PVDF‐HFP/fluoro‐surfactant	EMIMOTF	p	38.3	0.8	0.20	1.6	—	2.3	[53]
PDMAPS	EMIMDCA	p	12.8	31.0	0.13	0.01	—	—	[56]
PVDF‐HFP/PEG	EMIMTFSI	p	14.0	0.2	—	—	—	—	[57]
P(([EMIM][SPA])‐*co*‐MA)	EMIM	p	5.8	0.04	0.20	0.001	—	0.002	[58]
PSS‐Na	Na	p	4.0	1.0	0.49	0.02	—	0.01	[96]
Ligin/PVA	KOH	p	5.7	5.2	0.20	0.2	—	0.3	[157]
PAAm‐alginate/PEG	EMIMBF_4_	n	19.3	12.5	0.38	—	0.003	—	[47]
PVA/TOBC	NaOH	n	20.7	0.06	—	0.3	—	—	[48]
PVDF‐HFP/TPFPB	NaTFSI	n	6.0	0.09	0.19	—	—	0.04	[49]
PEO	LITFSI /EMIMBF_4_	n	15.0	0.2	0.11	0.04	—	—	[50]
PVA	NaOH	n	37.6	0.007	0.42	—	—	—	[54]
Bacterial cellulose	CaCl_2_/NaCl	n	27.2	20.4	0.32	15.1	—	—	[55]
PMACP	EMIMTFSI	n	2.8	1.4	0.10	2.4	—	—	[56]
PVDF‐HFP	EMIMTFSI	n	4.0	0.1	0.14	—	—	—	[57]
P(([APTA][TFSI])‐*co*‐MA)	TFSI	n	4.2	0.03	0.12	0.0005	—	0.001	[58]

### 2.3 Working Mechanism of the Thermogalvanic Effect: Redox Reaction

In the TG effect, electrons are transferred to the electrode via temperature‐dependent reversible redox reactions (Figure 2c,d) [64, 65, 66, 67]. In the absence of a temperature gradient, the reversible redox reaction remains in equilibrium. However, when a temperature gradient is applied, this equilibrium is disrupted, favoring either the reduction or oxidation reaction. The Gibbs free energy change (Δ*G*) resulting from the redox reaction is described as follows, Equation (10) [123]:

(10) $$\Delta G=\Delta G^{\circ }+RTln\frac{a_{Red}}{a_{Oxi}}$$

where *R* and *a_i_* are the gas constant and activity of species *i*.

Moreover, the relation between Δ*G* and electric potential (*E*) is expressed as Equation (11):

(11) ΔG=−nFE

where *n* and *F* are the number of electrons involved in the redox reaction and the Faraday constant. The thermodynamic expression of *E* is obtained by combining Equations (10) and (11):

(12) $$E=-\frac{\Delta G^{\circ }}{nF}-\frac{RT}{nF}ln\frac{a_{Red}}{a_{Oxi}}$$

In LS‐ionic conductors, the *a_i_* of the redox couples is approximately constant and similar at both electrodes. Thus, the *a_i_* term becomes negligible, and the *E* simplifies to Equation (13):

(13) $$E=-\;\frac{\Delta G^{\circ }}{nF}=-\frac{\Delta H^{\circ }-T\Delta S^{\circ }}{nF}$$

Assuming that Δ*H* ^°^ and Δ*S* ^°^ are temperature‐independent around room temperature, the *E* depending on *T* (*α*) is defined as follows, Equation (14) [123]:

(14) $$\alpha =\frac{\partial E}{\partial T}=\frac{\Delta S^{\circ }}{nF}\;$$

Consequently, the *α* of the TG effect in LS‐ionic conductors is determined by the Δ*S* of the redox couples [123, 124]. Furthermore, the TG effect is classified as “p‐ or n‐type” according to the prevailing direction of the reversible reaction. In general, the redox reaction proceeds toward increasing the entropy at the hot‐side electrode, whereas the redox reaction proceeds to a relatively decreasing entropy at the cold‐side electrode [117]. Redox couples that have a decreasing (increasing) entropy during a reduction reaction typically exhibit “p (n)‐type” TG effect. Accordingly, the selection of redox couples is crucial for determining the sign and magnitude of the TG [123].

On the contrary, in QSS‐ionic conductors, strong interaction between the polymer and redox couples leads to a non‐uniform distribution, which alters the *a_Red_*/*a_Oxi_*. Hence, the *a_i_* term cannot be neglected [67, 70, 93, 147]. For example, some researchers have endeavored to enhance the *a_Red_*/*a_Oxi_* by leveraging interactions between the polymer matrix and specific redox couples, resulting in significant improvements in *α* [64, 70, 93, 147]. Consequently, a larger Δ*S* in the redox reaction and *a_i_* ratio result in a higher potential difference between the two electrodes, increasing *α* in QSS‐ionic conductors (Table 2) [38, 63, 64, 65, 66, 67, 68, 69, 70, 71, 72, 73, 74, 75, 76, 77, 78, 79, 80, 81, 82, 83, 84, 85, 86, 87, 88, 89, 90, 91, 92, 93, 147, 149, 193].

**TABLE 2:** Comparison of TG effect (*α*, *σ_i_*, thermal conductivity (*κ*), power factor (*PF*), power density, figure of merit (*ZT*), *η_r_*) of reported TG cells based on polymers.

Polymer	Redox couple	Type	*α* (mV K^−1^)	*σ_i_* (S m^−1^)	*κ* (W m^−1^ K^−1^)	*PF* (mW m^−1^K^−2^)	Power density (W m^−2^)	*ZT*	*η_r_* (%)	Refs.
PAAm/SA	Fe(CN)_6_^3‐/4−^	p	4.4	10.5	0.6	0.2	0.4	0.11	3.140	[38]
PVA	Fe(CN)_6_^3‐/4−^	p	6.5	12.0	0.5	0.2	1.7	0.1	2.7	[63]
P(AAm‐*co*‐AMPS)	Fe(CN)_6_^3‐/4−^	p	1.6	12.0	0.1	—	0.05	0.07	1.4	[64]
PVA/PAAm	Fe(CN)_6_^3‐/4−^	p	1.5	1.7	1.0	0.004	—	—	—	[66]
PNAGA‐F68	Fe(CN)_6_^3‐/4−^	p	2.2	7.0	—	—	0.3	—	—	[69]
Gelatin	Fe(CN)_6_^3‐/4−^	p	17	1	0.2	—	0.004	—	0.006	[71]
PAA	Fe(CN)_6_^3‐/4−^	p	8.2	4.7	0.5	—	2.4	0.17	4.91	[72]
Bacterial Cellulose	Fe(CN)_6_^3‐/4−^	p	1.5	26.1	—	60.4	0.02	—	—	[74]
PAAm	Fe(CN)_6_^3‐/4−^	p	1.4	1.3	1.1	0.006	0.05	0.002	—	[76]
PVA	Fe(CN)_6_^3‐/4−^	p	1.2	0.6	1.9	—	—	—	—	[77]
PVA	Fe(CN)_6_^3‐/4−^	p	1.5	2.6	0.4	—	—	—	—	[78]
PSBMA	Fe(CN)_6_^3‐/4−^	p	3.5	10.0	—	—	0.002	—	—	[147]
Gelatin/Glutaraldehyde	Fe(CN)_6_^3‐/4−^	p	24.7	—	0.2	2.2	5.1	—	—	[90]
Gelatin/Betaine	Fe(CN)_6_^3‐/4−^	P	2.2	4.0	0.4	0.02	0.5	—	—	[79]
PVA	Fe(CN)_6_^3‐/4−^	P	7.2	13.0	0.4	—	8.5	—	—	[80]
PVA	Fe(CN)_6_^3‐/4−^	P	1.9	2.7	0.4	0.01	0.003	—	—	[82]
P(AA‐*co*‐HEA)/hPEI	Fe(CN)_6_^3‐/4−^	P	2.1	0.05	—	—	—	—	—	[84]
PVA/PAAm	Fe(CN)_6_^3‐/4−^	P	3.9	14.7	—	0.1	—	—	—	[86]
PAAm/Alginate	Fe(CN)_6_^3‐/4−^	P	1.8	2.8	—	0.007	0.01			[87]
PAAm/Gelatin	Fe(CN)_6_^3‐/4−^	P	4.0	1.2	—	0.004	1.0	0.009		[88]
PEDOT:PSS/PVA	SO_4/3_^2−^	p	1.63	2.9	0.8	—	—	—	—	[65]
PNIPAM	I^−^/I_3_^−^	p/n	1.9 /0.7	0.1 /0.1	1.2/0.5	—	—	—	—	[70]
PAAm‐*co*‐AAK	BQ/HQ	p	25.9	7.4	0.5	5.0	0.01	0.003	—	[193]
P(NIPAM‐*co*‐AA) Nanoparticle	BQ/HQ	p/n	6.7 /8.9	0.04 /1.5	—	—	—	0.0009 /0.05	—	[91]
PVA/PDMS	Fe^2+/3+^	n	1.5	1.5	1.1	—	—	0.001	—	[67]
PAAm/Alginate	Fe^2+/3+^	n	1.4	4.0	—	0.001	—	0.003	—	[68]
Chitosan Guanidine/ Methyl Cellulose	Fe^2+/3+^	n	7.2	12.8	0.5	—	4.5	—	—	[73]
PVA/PEG	Fe^2+/3+^	n	0.9	0.2	—	—	0.01			[83]
PAAm	Fe^2+/3+^	n	1.7	6.5	0.9	0.003	0.03	0.001	—	[76]
PVA	Fe^2+/3+^	n	1.0	1.0	1.9	—	—	—	—	[77]
PAM‐*co*‐ DMAEA‐Q	Fe^2+/3+^	n	1.9	0.05	0.4	0.004	0.2	0.003	—	[93]
Alginate	Fe^2+/3+^	n	3.6	1.8	—	—				[81]
PVA	Cu^0/2+^	n	0.7	0.1	—	—	0.0005	—	—	[75]
PVA/Carrageenan	I^−^/I_3_^−^	n	1.1	0.03	0.3	—				[85]
PLAM	BQ/HQ	n	4.3	1.0	0.01	—	0.03	—	1.1	[149]

## 3 Hybrid Transport Strategies in Ionic Thermoelectrics

### 3.1 Synergistic Effect Integrating TD and TG Effects for High Thermopower

**FIGURE 3:**
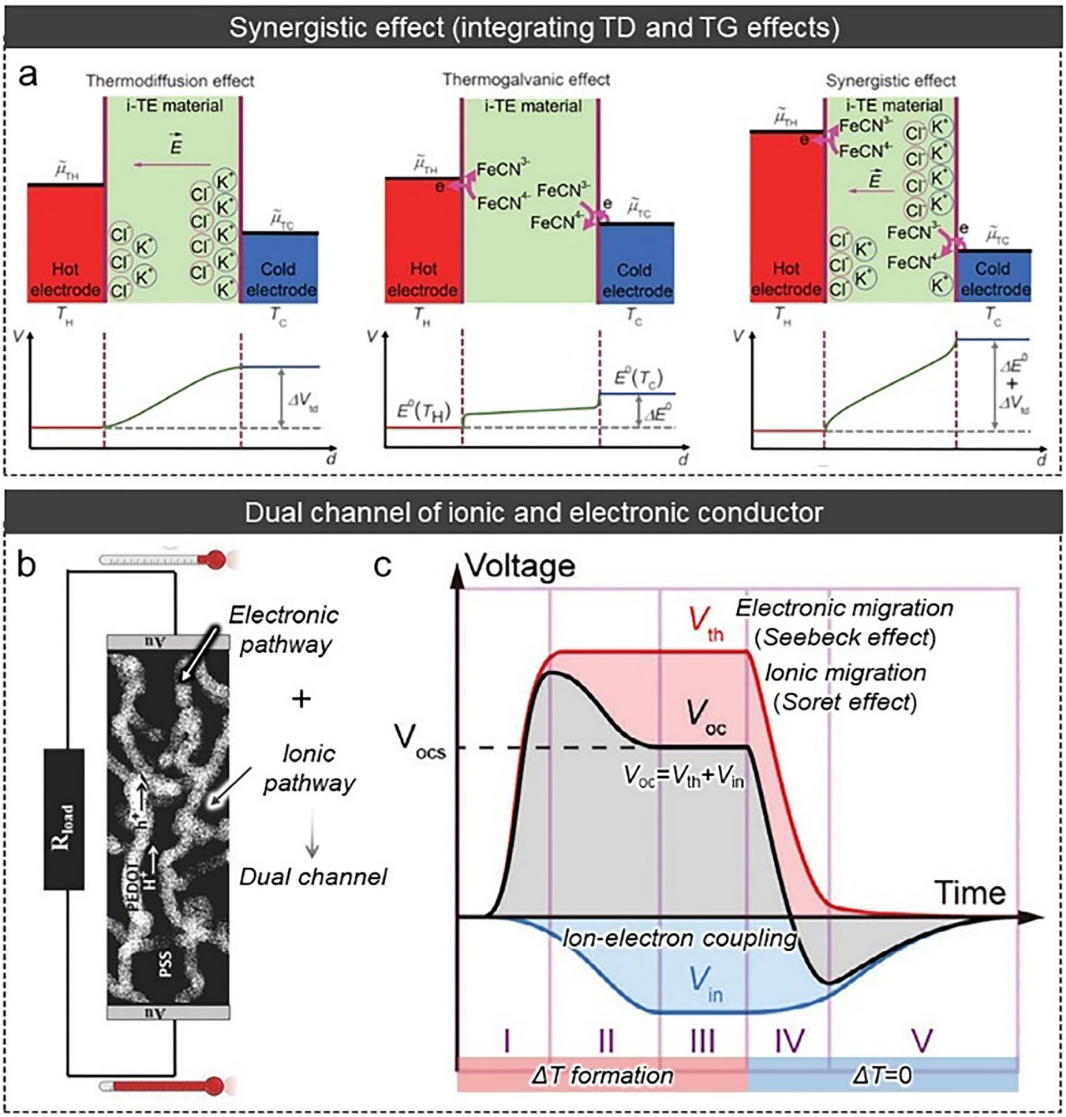
(a) Working principle and voltage profile of synergistic effect. Reproduced with permission [71]. Copyright 2020, The American Association for the Advancement of Science (AAAS). (b) Illustration of the dual channel (ionic‐electronic) conductors pathway. Reproduced with permission [125]. Copyright 2016, Wiley‐VCH. (c) Working principle and voltage profile of dual (ionic‐ and electronic‐) conductors based on iTEs. Reproduced with permission [126]. Copyright 2025, Wiley‐VCH.

Although the TG effect allows for continuous energy generation, it has less *α* than the TD effect. Thus, Han et al. attempted a synergistic effect strategy: integration between the TD effect (KCl as mobile ions) and TG effect (Fe(CN)_6_^3−/4−^ as redox couples) in a gelatin matrix (Figure 3a) [71]. They analyzed the contribution of TD and TG effects separately via an isothermal system, and based on this analysis, they derived the *α* via the synergistic effect as follows 15:

(15) $$\alpha =-\alpha_{Red}+\alpha_{td}\left(K^{+}-Fe{{\left(CN\right)}_{6}}^{3-/4-}\right)+\alpha_{td}\left(KCl\right)+\alpha_{td}\left(Gelatin\right)$$

where *α_Red_* is the TG effect due to the redox reaction between redox couples, *α_td_* denotes the TD effect attributed to mobile ions, and *α_td_* (*Gelatin*) indicates the intrinsic *α* from the polymer matrix.TD and TG cells exhibited 6.7 and 4.8 mV K^−1^ *α*, respectively. Meanwhile, the synergistic effect showed 12.7 mV K^−1^ *α* owing to the coupling of the TD and TG effects.

### 3.2 Coupled Ionic‐Electronic Transport Enabling Ion‐to‐Electron Energy Transduction

iTEs generate TE outputs via ionic transport driven by temperature gradients. While these systems enable significant *α* (TD effect) or inherently facilitate continuous current through redox processes (TG effect), purely ionic systems are inherently limited in how thermodynamic driving forces are converted into usable electrical power, frequently encountering significant internal resistance and kinetically restricted current extraction in practical devices [125, 126, 127, 128].

To overcome these fundamental energy conversion constraints, rather than merely transport limitations, mixed ionic‐electronic conductors (MIECs) have been devised to change the working framework that facilitates ion‐to‐electron conversion via coupled ionic‐electronic transport pathways. (Figure 3b) [125]. MIECs, rather than merely improving transport, shift TD‐induced ionic polarization into electronic drift currents by converting ionic thermodynamic factors directly into electronic charge transfer [129]. This hybrid conductor fundamentally changes the working mechanism of iTEs by providing a direct energy conversion pathway wherein TD‐induced ionic polarization itself promotes electronic transport [130].

In MIECs, ion transport driven by a temperature gradient generates a TE potential in the form of an internal electric field. The ion‐induced electric field serves as a stimulant for the migration of electrons within electronically conductive pathways. Consequently, electrons respond to the ionic thermodynamic potential via ion‐electron coupling, facilitating the electronic current flow in response to ion‐induced thermoelectric polarization (Figure 3c). While continuous electronic extraction of ion‐induced electric fields reduces the ionic thermodynamic potential, ion‐electron coupling effectively converts this driving force into continuous electronic current, overcoming constraints of ion‐dominated transport [129, 131]. This approach allows for rapid electrical response and steady power extraction even under more complex thermal gradients, hence expanding the practical applications of iTEs beyond static thermal conditions [131].

The intensity and behavior of ion‐electron coupling are significantly affected by the relative levels of ionic and electronic conductivities and the spatial arrangement of ionic and electronic pathways. In slow electron transport, MIECs serve in an ionic‐dominant system, where electrons primarily contribute to charge extraction without reducing the ionic potential. In contrast, when electron conduction becomes comparable to ion conduction, pronounced electrostatic screening and dynamic coupling effects arise, resulting in more balanced mixed‐transport behavior with enhanced current but decreased thermo‐voltage (Figure 3c) [126, 127, 128]. This coupling system demonstrates MIECs as fundamental design platforms for regulating energy conversion pathways in iTEs, rather than simply enhancing transport [130].

## 4 Molecular‐Scale Engineering for Improving the Ionic Thermoelectric Performance

### 4.1 Interaction Between the Polymer Matrix and Mobile Ions for Selective Ion Transport in the TD Effect

QSS‐ionic conductors have relatively less solvent compared to LS‐ionic conductors, which reduces the influence of mobile ions on the hydration shell [132, 133, 134]. Thus, the impact of size and weight on the migration of mobile ions intensifies, resulting in an enlarged difference in diffusivity between cations and anions [135, 136]. Moreover, functional groups of the polymer are involved in additional interactions with mobile ions and the other polymers in the systems. The interaction further promotes the difference in diffusivity between cations and anions [50, 121].

**FIGURE 4:**
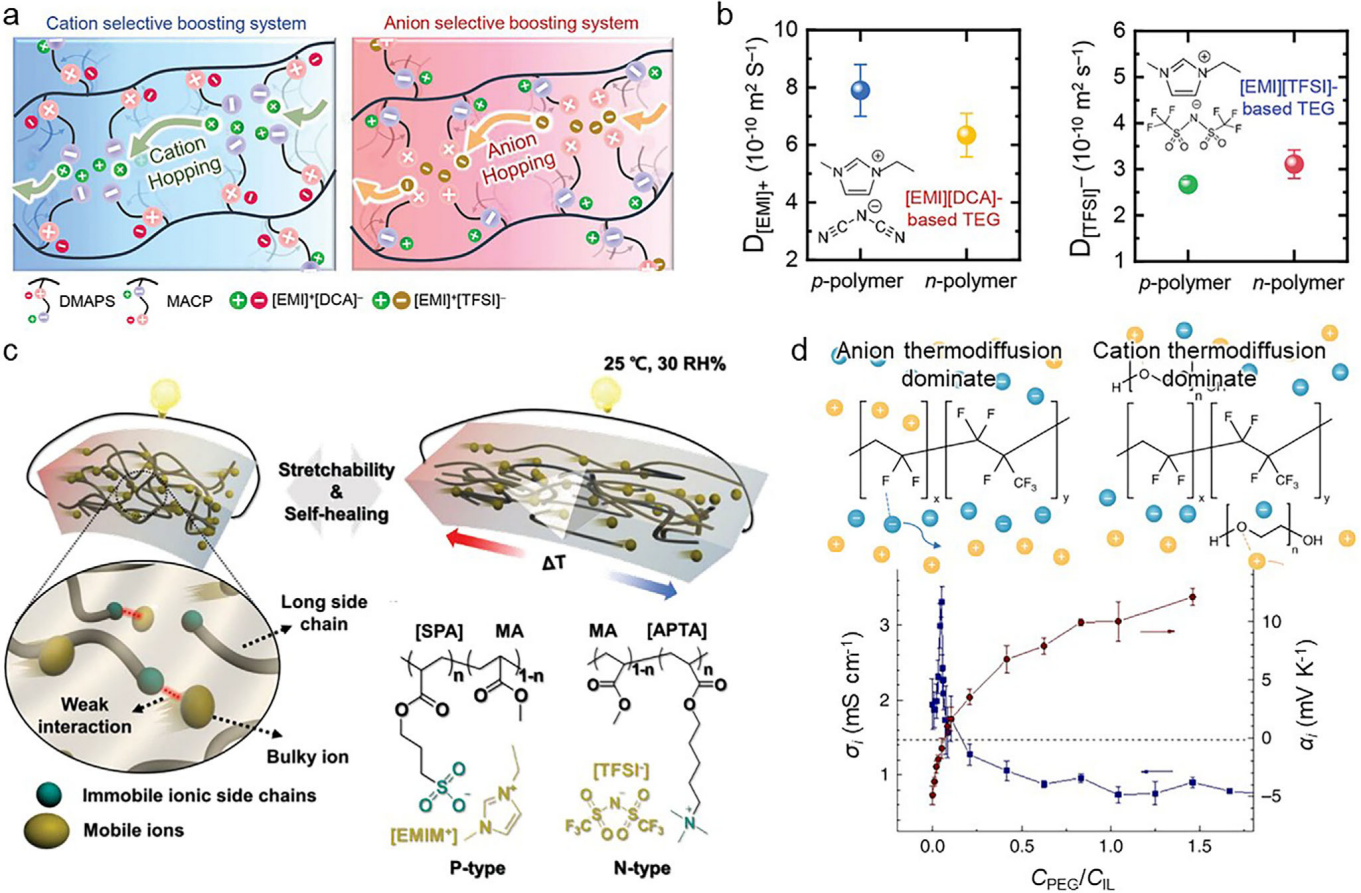
(a) Schematic diagram of regulating ion diffusivity through the zwitterionic polymers. (b) Ionic liquid diffusivity in zwitterionic polymers. Reproduced with permission [56]. Copyright 2023, Wiley‐VCH. (c) Illustration of p‐ and n‐type single ionic polymer. Reproduced with permission [58]. Copyright 2023, Wiley‐VCH. and (d) Diagram of mobile ions transport and converting TD effect depending on the PEG. Reproduced under the terms of the CC‐BY 4.0 license [57]. Copyright 2019, The Authors, Springer Nature.

Specific functional groups (e.g., ionic moiety and polar moiety) in the polymer selectively interact with mobile ions, boosting the transport of cations (anions) while impeding the transport of anions (cations) [137, 138]. The selective interactions with mobile ions enhance the diffusivity difference between cations and anions, hence improving the accumulation of ions at the electrode interface, which forms an EDL, which determines the magnitude of the TD cells [139, 140]. For example, Ho et al. reported polymers with zwitterionic groups at the side chain [56]. The zwitterion facilitated the dissociation between cation and anion by the electrostatic interaction between the polymer and mobile ions. In addition, the ion moiety at the end of the side chain increased counter‐ion transport, whereas the ion moiety near the polymer backbone constrained counter‐ion transport (Figure 4a). Thus, the sign of the TD effect was determined by the distance of the ionic moiety from the polymer backbone. To demonstrate the difference in ion transport, they compared the diffusivity of cations and anions in zwitterionic polymers (Figure 4b). 1‐Ethyl‐3‐methylimidazolium (EMIM^+^) exhibited 7.9 × 10^−10^ (in p‐type) and 6.3 × 10^−10^ m^2^ s^−1^ (in n‐type), respectively. Conversely, bis(trifluoromethylsulfonyl)imide (TFSI^−^) was 2.67 × 10^−10^ (in p‐type) and 3.11 × 10^−10^ m^2^ s^−1^ (in n‐type), respectively. Consequently, the cation exhibited faster transport in the p‐type polymer compared to the n‐type polymer. Meanwhile, the anion was transported more rapidly in the n‐type polymer compared to the p‐type polymer. Thus, the *α* of the p (n)‐type polymers were 12.8 and −2.8 mV K^−1^, respectively. Furthermore, they confirmed the above results for various ionic liquids. Therefore, the effects of ion dissociation and transport in the zwitterionic polymer were demonstrated.

In addition, Kim et al. synthesized polymers with single ionic groups, enabling the transport of specific ions (either cations or anions) to maximise the cation and anion diffusivity difference [58]. The polymers with anionic (cationic) groups in the side chains exhibited only cation (anion) transport (Figure 4c). Moreover, polymers contained counter‐ions as mobile ions. Thus, the electrical and TE properties of the single ionic polymers were determined by regulating the number of monomers with ionic side chains. Consequently, the p (n)‐type polymers exhibited α of 5.74 (−4.18) mV K^−1^. Furthermore, they used chaotropic mobile ions, which reduced the hygroscopic properties of the polymers despite the presence of ionic moieties. Thus, they demonstrated single ionic polymers with significant repeatability regardless of surroundings.

Furthermore, the incorporation of the polar polymer altered the diffusivity of cations and anions in the previous QSS‐ionic conductors, thereby converting the sign of the TD effect [57, 141]. Zhao et al. utilized PEG in poly(vinylidene fluoride‐*co*‐hexafluoropropylene) (PVDF‐HFP) [57]. In the PVDF‐HFP matrix, anions exhibited faster migration compared to the cations. However, ether groups in PEG interacted with cations, boosting the cation migration and converting the sign of TE effect from “n‐type (−4 mV K^−1^)” to “p‐type (14 mV K^−1^)” (Figure 4d).

### 4.2 Interaction Between the Polymer and Specific Redox Materials for High Entropy Differences in the TG Effect

**FIGURE 5:**
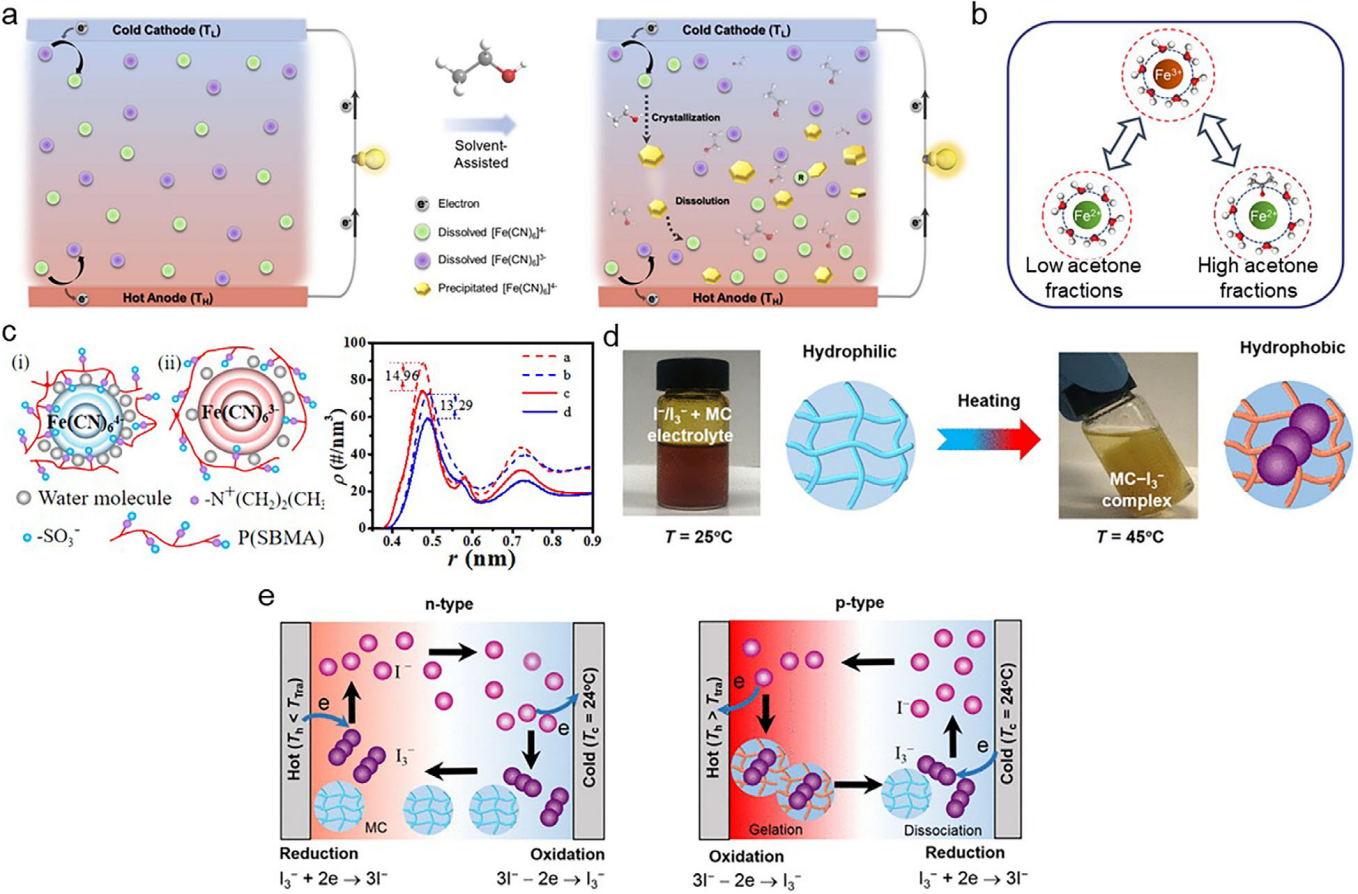
(a) Comparison of redox couples depending on the solvent‐assisted. Reproduced with permission [145]. Copyright 2025, Elsevier. (b) Hydration shell change between the redox couples according to the solvent fraction. Reproduced with permission [146]. Copyright 2023, Wiley‐VCH. (c) Radical density profiles of hydrated Fe(CN)_6_^4−^ (a and c) and Fe(CN)_6_^3−^ (b and d) in pure water (a and (b) and the P(SBMA) hydrogel (c and d) by molecular dynamic simulations and the solvation structure of Fe(CN)_6_^3‐/4−^ in the P(SBMA). Reproduced with permission [147]. Copyright 2024, Royal Society of Chemistry. (d) Photographic image of thermosensitive MC before and after applying heat. (e) Converting the redox reaction (sign of the TG effect) depending on the temperature. Reproduced under the terms of the CC‐BY 4.0 license [150]. Copyright 2022, The American Association for the Advancement of Science (AAAS).

In the TG effect, electrical energy is generated by the temperature‐dependent reversible reaction. The magnitude of energy is determined by the entropy difference between the redox couples [142, 143]. Therefore, various strategies have been explored to enhance the entropy difference between redox couples [142, 143, 144, 145, 146]. The entropy difference in various redox couples arises from Coulombic interactions between the charged species and their solvation shells in LS‐TG cells [144, 145, 146]. Thus, to enhance *α* in LS‐TG cells, researchers used various solvents for regulating the hydration shell and configuration (interaction between the redox couples and solvent) or adjusted counter‐ions for controlling the Coulombic strength (interaction between redox couples and counter ions) (Figure 5a,b) [145, 146]. However, QSS‐TG cells exhibit another interaction between the redox couples and the polymer matrix. Thus, some researchers attempted a selective interaction strategy between the polymer matrix and specific redox materials for enhancing the entropy difference between redox couples [147, 148]. Gao et al. utilized copolymers with chaotropic cationic side chains to selectively modify the configuration of Fe^3+^, increasing the entropy difference between the oxidant and reductant. The *α* was remarkably enhanced, reaching up to −2.02 mV K^−1^. Moreover, Liu et al. utilized a zwitterionic (sulfobetaine) polymer [147]. The cation moieties in sulfobetaine selectively interacted with Fe(CN)_6_^4−^, leading to the rearrangement of the hydration structure (Figure 5c). This led to an increase in *α* to 2.04 mV K^−1^.

### 4.3 Interaction Between the Polymer Matrix and Specific Redox Materials for the Concentration Gradient in the TG Effect

In addition, reversible redox reactions are affected by the *a_i_* of oxidant and reductant [38, 63, 64, 65, 66, 67, 68, 69, 70, 71, 72, 73, 74, 75, 76, 77, 78, 79, 80, 81, 82, 83, 84, 85, 86, 87, 88, 89, 90, 91, 92, 93, 142]. Therefore, some researchers regulated the concentration of specific redox couples through selective interaction [150, 151]. In particular, thermosensitive polymer matrices were utilized for establishing the concentration gradient across the systems. For example, Han et al. used thermosensitive methyl cellulose (MC) [150]. MC with I^−^/I_3_^−^ exhibited the “n‐type” TG effect under transition temperature (∼56°C). However, over the transition temperature, the hydrophobic properties were prominent due to the methyl group in MC, leading to the selective interaction with I_3_^−^ (more hydrophobic than I^−^) (Figure 5d). The capturing caused the free I_3_^−^ concentration gradient depending on the temperature gradient, which converts the reversible reaction direction. Consequently, it exhibited “p‐type” TG effect above the transition temperature (Figure 5e).

### 4.4 Interaction Between the Polymer Matrix and Mobile Materials for High Thermopower in the Synergistic Effect

**FIGURE 6:**
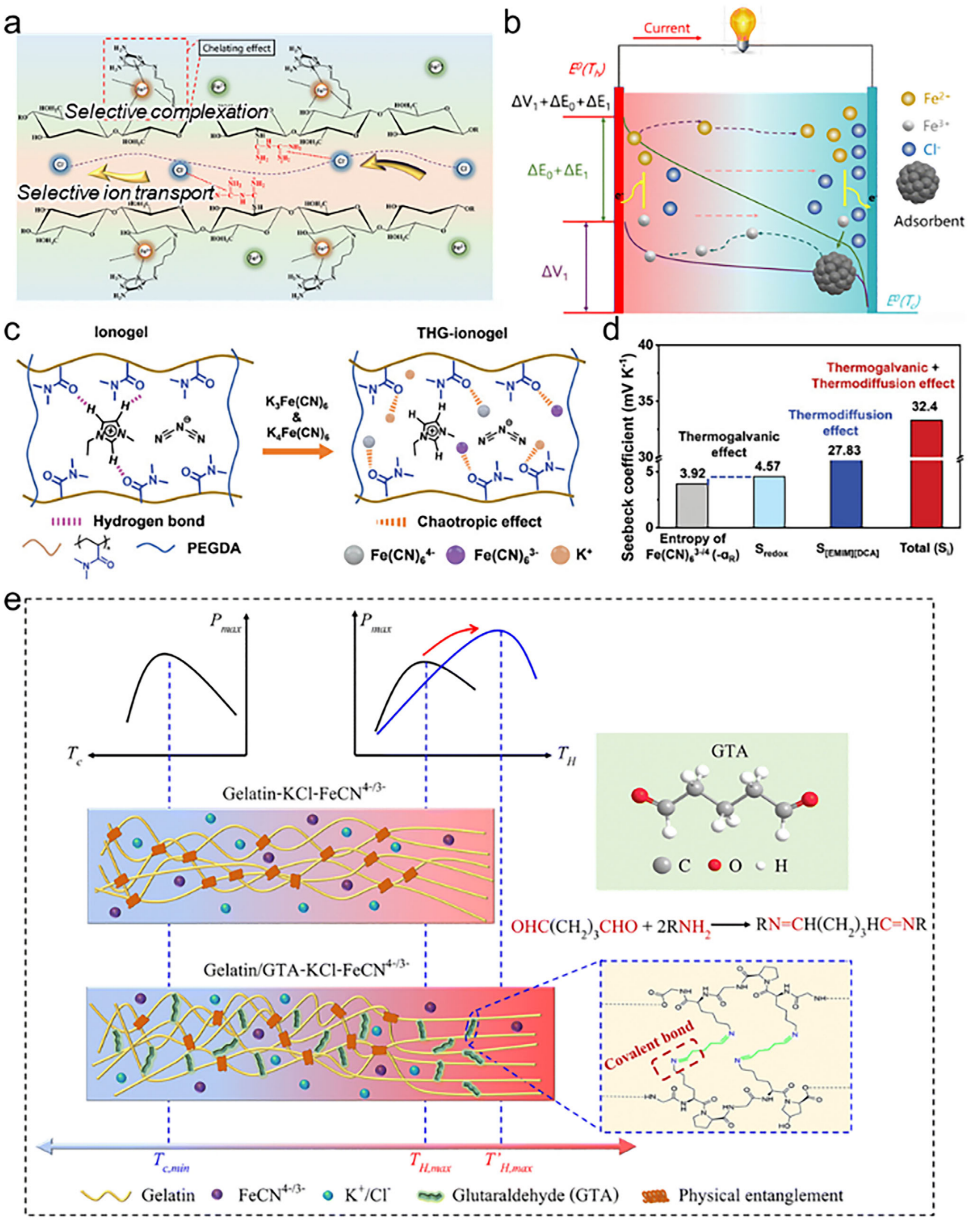
Synergistic effect by (a) selective ion transport and (b) complexation of redox materials at the cold side. Reproduced with permission [73]. Copyright 2024, Royal Society of Chemistry. (c) Schematic diagram of the thermal diffusion and migration model of ions in ionogels without/with redox materials. (d) Components of the total *α* of Fe(CN)_6_^3‐/4−^ ionogel. Reproduced with permission [152]. Copyright 2024, Wiley‐VCH. (e) Illustration of synergistic effect and improvement of working temperature range by cross‐linked polymer matrix using GTA. Reproduced with permission [153]. Copyright 2022, Royal Society of Chemistry.

Many investigators have optimized the *α* of synergistic cells by leveraging the interaction between the polymer matrix and mobile materials. These interactions regulate ion transport, selective ion association/dissociation, and behavior of redox couples, thereby modulating the contributions of TD and TG effects [73, 152, 153]. Hu et al. designed the grafting of biguanide salts into the chitosan matrix to accelerate separation between cations and anions and selective ion transport, which improved the *σ_i_* up to 12.78 S m^−1^ (Figure 6a). Meanwhile, the melamine in the polymer formed selective complexes with Fe^3+^ at the cold side by the adsorption effect (Figure 6b). Through complexation, the entropy and concentration differences between the redox couples were simultaneously amplified, leading to a significant increase in *α* from −1.04 to −7.24 mV K^−1^.

Yang et al. enhanced the *α* (32.4 mV K^−1^) via a synergistic effect and chaotropic effect [152]. They used EMIM^+^ dicyanamide (DCA^−^), Fe(CN)_6_^3−/4−^, and poly(N,N‐dimethylacrylamide) (PDMAA) as the mobile ions, redox couples, and polymer matrix, respectively. EMIM^+^ and amide groups in the PDMAA interacted via hydrogen bonds, which hindered the transport of EMIM^+^. However, when Fe(CN)_6_^3−/4−^ was incorporated into the ionogel (PDMAA/EMIM^+^DCA^−^), it disrupted the hydrogen bonds, leading to new interactions between the redox couples and PDMAA because of the chaotropic effect (Figure 6c). The release of EMIM^+^ increased ion mobility, contributing to an increase in *α*. They then analyzed the effect of Fe(CN)_6_^3−/4−^, EMIM^+^DCA^−^, and PDMAA on the *α* (Figure 6d). The *α* of the Fe(CN)_6_^3−/4−^ ionogel was higher than that of the ionogel and Fe(CN)_6_^3−/4−^ PDMAA.

The gelatin matrix relied on polymer chain entanglement, making it prone to structural collapse at high temperatures. The collapse of the structure increased internal resistance. Thus, the thermophoresis was suppressed, and the current and output power density were decreased. To improve this, Li et al. added glutaraldehyde (GTA) as a cross‐linker within the gelatin polymer matrix (Figure 6e) [153]. Chemical cross‐linking via GTA reinforced the polymer structure, extending its working temperature range. Additionally, GTA selectively interacted with Fe(CN)_6_^3−/4−^, boosting both mobility and entropy differences in the redox couples. Consequently, they reported an exceedingly high *α* (24.7 mV K^−1^).

## 5 Micro‐Scale Engineering for Improving the Ionic Thermoelectric Performance

### 5.1 Phase Separation Depending on the Temperature for Enlarging the Diffusivity Difference in the TD Effect

**FIGURE 7:**
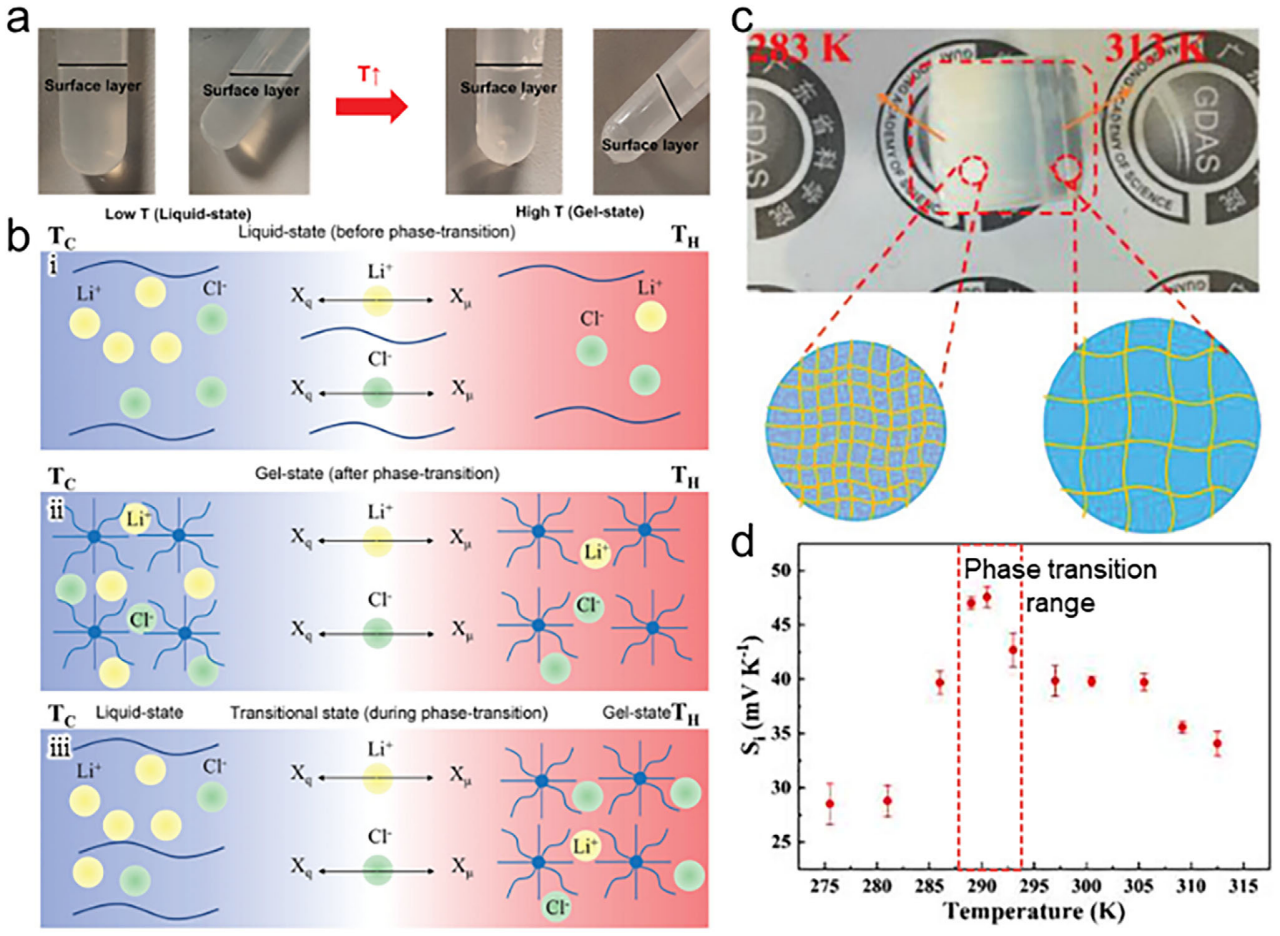
(a) Liquid‐to‐gel phase transition of LiCl/poloxamer 407 solution. (b) Schematic diagram of the diffusion process of cations and anions under temperature gradient, (i) liquid‐state, (ii) quasi‐solid‐state, and (iii) liquid‐to‐gel phase transition‐state. Reproduced with permission [37]. Copyright 2022, Wiley‐VCH. (c) Photographic image of IPN polymer under ∆*T*. (d) *α*‐temperature relationship of IPN polymer under a ∆*T* = 5 K. Reproduced with permission [40]. Copyright 2023, Wiley‐VCH.

Mobile ions migrate to interstitial regions in the polymer matrix and arrive at the electrode interface [154]. Therefore, regulating the interstitial regions in the polymers contributes to the diffusivity difference [155]. Therefore, some investigators have tried to improve the diffusivity difference of mobile ions by controlling the interstitial region [36, 40]. Liu et al. analyzed the phase transition effect with poloxamer 407, a polymer comprised of a triblock structure with hydrophilic (PEG) and hydrophobic (poly(propylene glycol) (PPG)) parts [37]. PPG parts in the poloxamer 407 dehydrate with increasing temperature, which form spherical structures (gel‐state). Thus, poloxamer 407 exhibits a sol‐to‐gel phase transition at high temperature (Figure 7a). In the sol‐state of poloxamer 407, the solvent in the polymer had a considerable influence on the mobility of both cations and anions, contributing to their similar diffusivity (Figure 7bi). However, in the gel‐state, the size of the ions significantly affected their diffusivity, allowing for an increased difference in diffusivity between the cations and anions (Figure 7bii). To enhance the difference in diffusivity between the cations and anions and the potential difference between the electrodes, a sol‐gel phase transition strategy was attempted. The sol‐gel phase transition polymer increased the *α* by 6.5 times compared to the homogeneous sol and gel state (Figure 7biii).

Furthermore, Jiang et al. improved the phase transition approach by incorporating an interaction between the polymer and ions [40]. They designed an interpenetrating network (IPN) polymer using an acrylic acid‐acrylamide copolymer (P(AA‐AAm)) and carboxymethyl cellulose (CMC). The dense hydrogen bonds between the amide groups in P(AA‐AAm) and the carboxymethyl groups in CMC caused agglomeration, which was observed to be opaque at low temperature. However, the hydrogen bonds were easily broken at high temperature, leading to volume expansion and a transparent state (Figure 7c). Therefore, the transport of bulk SO_4_^2−^ was impeded, and a small H^+^ was freely transported at low temperature. Moreover, the carboxylate group in CMC promoted electrostatic repulsion with SO_4_^2−^, thereby restricting its mobility to the cold side. Consequently, the IPN polymer exhibited a higher *α* (40.60 mV K^−1^) across the temperature range that included the phase transition, compared to the range without phase transition effects (Figure 7d).

### 5.2 Aligned Polymer Matrix With Functional Group‐Induced Diffusivity Contrast in the TD Effect

Moreover, many investigators aligned the interstitial region in the polymer matrix (ion channel), which accelerates the ion migration (high *σ_i_*). It alleviated the self‐discharging process, which aids in a high concentration of mobile ions at the EDL. Hence, research has been undertaken to enhance *σ_i_* while increasing the diffusivity difference of mobile ions.

Some researchers have endeavored to adjust the interstitial region via ion channels of an aligned polymer matrix [59, 156]. Li et al. used aligned cellulose to enhance diffusion [156]. Moreover, the alcohol groups in cellulose were oxidized to carboxylic acid. The carboxylic acid groups promoted the dissociation between the cations (Na^+^) and anions (OH^−^) by interacting with the anions. Therefore, oxidized aligned cellulose exhibited rapid selective cation transport. They analyzed the *σ_i_* depending on the concentration. Oxidized aligned cellulose exhibited higher *σ_i_* (∼3.0 S m^−1^) compared to the bulk electrolyte (∼1.8 S m^−1^) at a broad range of concentrations. As a result, oxidized aligned cellulose dramatically increased the *α* up to 24 mV K^−1^.

**FIGURE 8:**
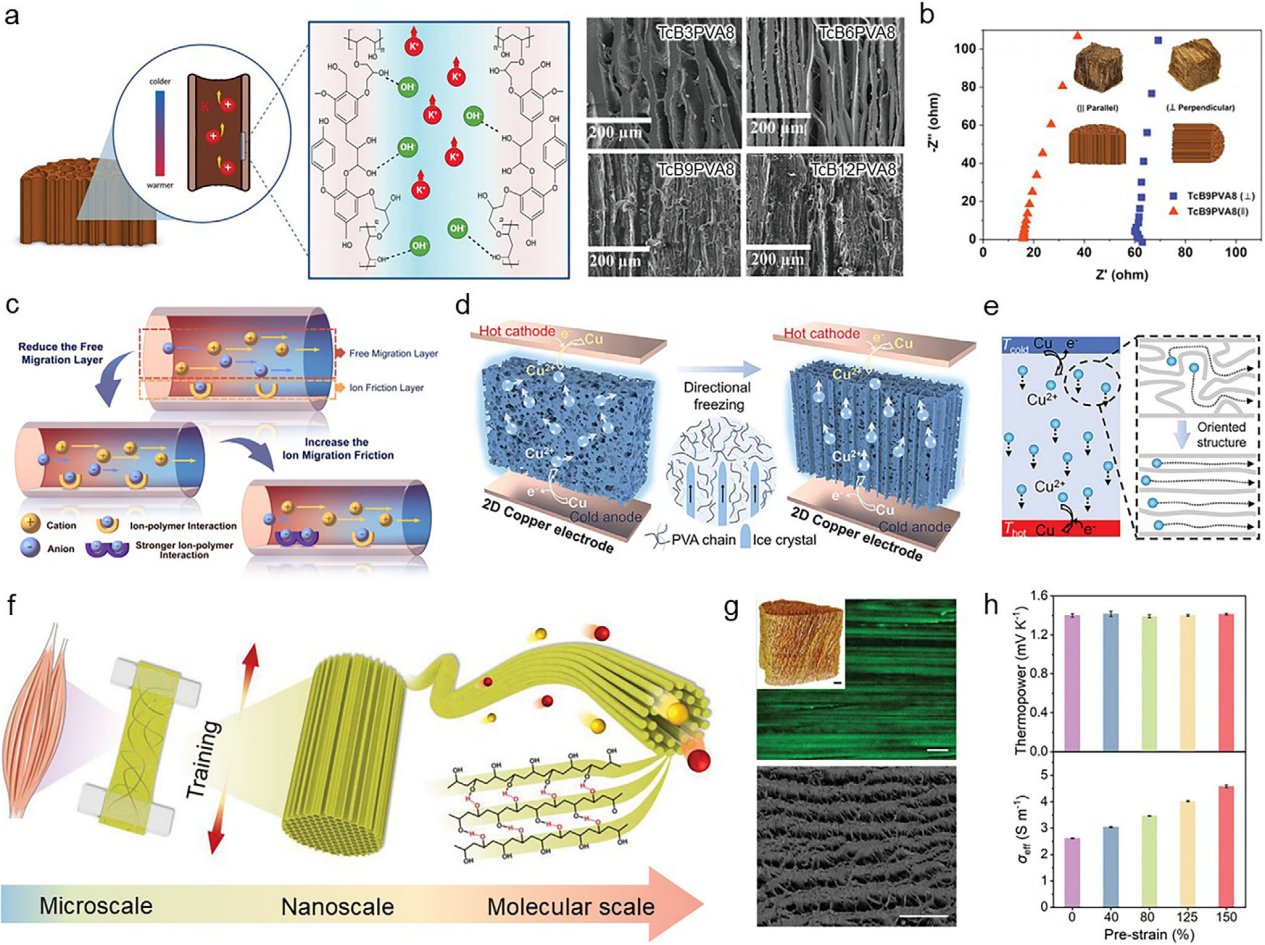
(a) Illustration of a selective cation transport in an aligned polymer. (b) *σ_i_* depending on the different directions. Reproduced with permission [157]. Copyright 2024, Wiley–VCH. (c) Diagram of thermal migration channel regulation. Reproduced with permission [154]. Copyright 2024, Elsevier. (d) Schematic of anisotropically structured polymer via the directional freezing‐thaw method. (e) Engineering of ion transport pathways through an anisotropic network. Reproduced with permission [75]. Copyright 2024, Wiley–VCH. (f) Schematic illustration of a mechanically trained anisotropic polymer. (g) In situ confocal and FESEM images of a mechanically trained anisotropic structure. (h) *α* and *σ_i_* of anisotropic TG cells as a function of pre‐strain magnitude. Reproduced with permission [158]. Copyright 2022, Wiley–VCH.

In addition, Muddasar et al. used vertically aligned lignin/ PVA for ion channels [59]. With the increase in lignin concentration in polymers, stable nano channels formed, whereas micro channels diminished correspondingly. However, excessive nano channels exhibited tortuous aligned channels. Furthermore, KOH electrolyte led to a negative charge surface of aligned channels by deprotonation of alcoholic and phenolic groups in lignin. This negative charge surface facilitated selective ion migration within nano‐sized channels (Figure 8a). Thus, lignin‐derived polymer exhibited high *σ_i_* (5.15 S m^−1^) and *α* (5.71 mV K^−1^). Moreover, they analyzed the *σ_i_* and *α* in perpendicular and parallel directions of lignin‐derived polymer. Perpendicular direction exhibited approximately 5 times lower *σ_i_* (1.12 S m^−1^) and *α* (1.09 mV K^−1^) (Figure 8b).

Furthermore, Cheng et al. tried to achieve selective ion transport with ion channels [154]. They regulated the ion channel size via water evaporation‐induced phase separation. Ion channels in a polymer matrix are divided into a free migration layer and an ion friction layer (Figure 8c). The free migration layer is located at the center of ion channels and has little effect on ion migration owing to the minimal polymer‐ion interaction. On the contrary, the ion friction layer is located at the wall in ion channels and binds nearby ions through interaction. Thus, they reduced the free migration layer for selective ion migration. Subsequently, they increased the binding strength with specific ions in the ion friction layer. Consequently, the strategy enhanced the *α* from 2.45 to 8.13 mV K^−1^.

### 5.3 Polymer Matrix Reorientation for Accelerating Redox Couples Transport in the TG Effect

Moreover, several researchers have tried to develop strategically ion channels in the polymer matrix through various methods [75, 158]. For example, Meng et al. introduced a directional freezing‐thaw method during the fabrication process of TG cells, which formed anisotropic structures within the polymer matrix [75]. In this process, the growth direction of ice crystals was confined to a specific orientation, serving as a template that promoted the crystallinity of PVA polymer chains via hydrogen bonds (Figure 8d). Consequently, the polymer chains aligned along the ice template, forming a dense crystalline structure with directional anisotropy (Figure 8e). In comparison to the isotropic TG cell, the anisotropic TG cell exhibited a 1.2‐fold enhancement in *σ_i_* (0.62 S m^−^^1^) and a similar *α* (0.62 mV K^−^^1^). In addition, optimization of the redox concentration resulted in an enhanced *σ_i_* of 0.8 S m^−1^ and *α* of 0.7 mV K^−1^.

Furthermore, Lei et al. were inspired by muscle growth through mechanical training to improve ion channels within the PVA matrix (Figure 8f). They applied various magnitudes of tensile strains after the freeze‐thaw method at the PVA [158]. The stretching reduced the distance between polymer chains, reinforced inter‐interaction, and strengthened the anisotropic structure (Figure 8g). Therefore, the further aligned polymer matrix not only maximized ion migration but also improved mechanical toughness (Figure 8f). As a result, this system achieved a high *σ_i_* of 4.6 S m^−1^ and *α* of 1.4 mV K^−1^ compared to ice‐templating samples (Figure 8h).

## 6 Macro‐Scale Engineering for Improving the Ionic Thermoelectric Performance

### 6.1 Specific Geometric Architectures for a Stable Thermal Gradient in the TD and TG Effects

**FIGURE 9:**
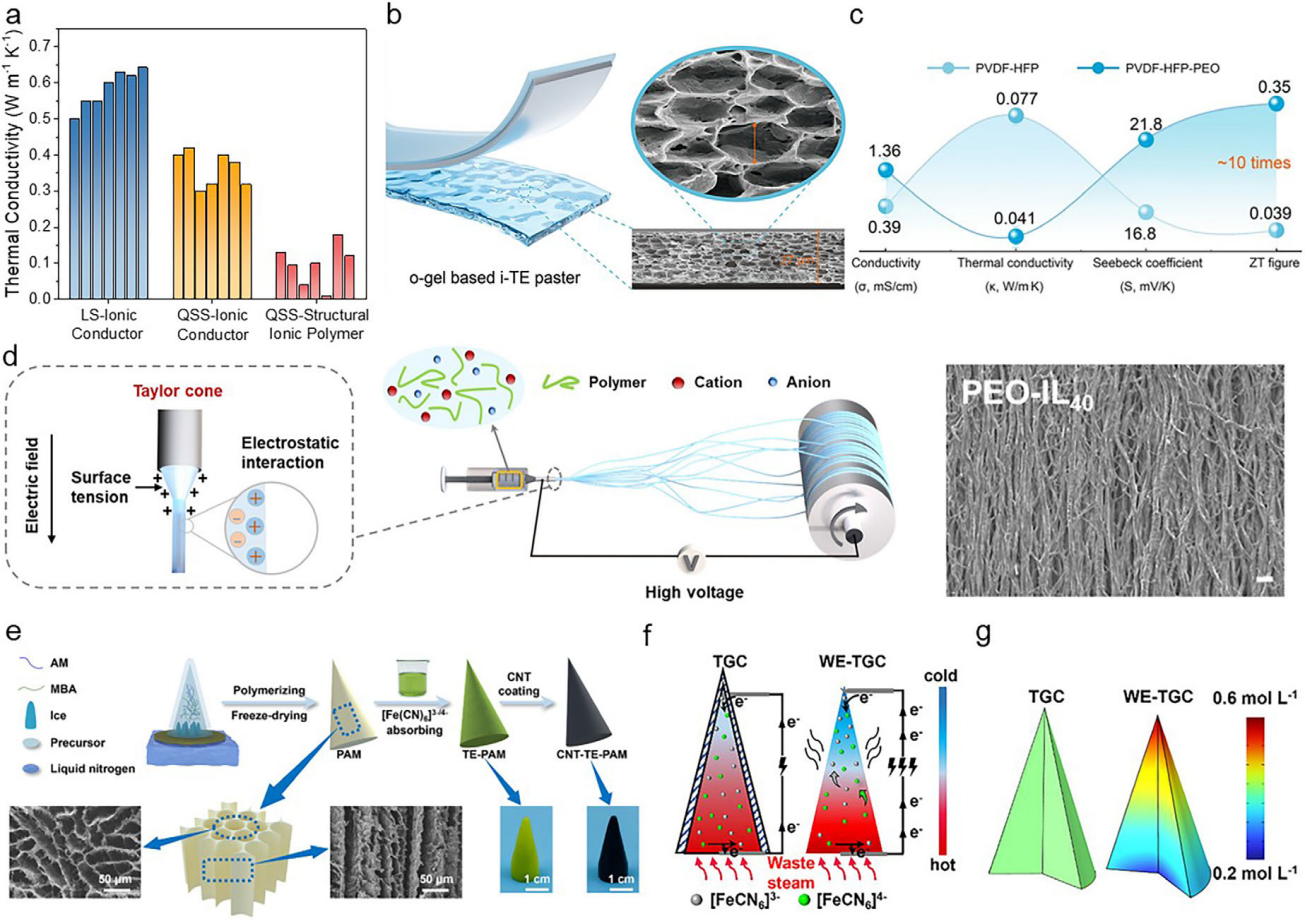
(a) *κ* of various ionic conductors (LS, QSS, and QSS structural & ionic polymer) [47, 54, 55, 56, 58, 61, 78, 79, 85, 149, 161, 162, 163, 164, 165, 166, 167, 168, 169]. (b) Illustration of onion epidermal cell‐like porous polymer frameworks. (c) *σ_i_*, *κ*, *α*, and *ZT* of porous structured TG cells. Reproduced with permission [168]. Copyright 2025, Springer. (d) Fabrication process of fibrous aligned TD cells. Reproduced with permission [169]. Copyright 2025, American Chemical Society. (e) Schematic diagram of the preparation process of conical TG cells. (f) Temperature gradient comparison between conventional TG cells and water‐evaporation TG cells (WE‐TG cells). (g) Numerical simulation of the concentration distribution in TG cells and WE‐TG cells after evaporation. Reproduced with permission [170]. Copyright 2025, Wiley–VCH.

The performance of iTE cells is primarily determined by *σ_i_*, *α*, and *κ* [159, 160]. Especially, low *κ* is crucial for sustaining a steady and high temperature gradient within the system, which promotes ion migration and redox reactions. However, *κ* is an intrinsic characteristic of the material, making it challenging to regulate (Figure 9a) [47, 54, 55, 56, 58, 61, 78, 79, 85, 149, 161, 162, 163, 164, 165, 166, 167, 168, 169]. While *κ* can be adjusted by controlling the crystallinity of materials, significant alterations are challenging. Consequently, numerous researchers have attempted to modulate *κ* via structural‐scale engineering [168, 169]. For example, Du et al. designed onion epidermal cell‐like structure polymer frameworks in the TG cells [168]. The framework‐embedded structure, supported by porous PVDF‐HFP/PEG membranes, enhanced performance by the thermal expansion effect (Figure 9b). The porous membrane structure contained an enormous amount of electrolyte, which enabled a higher ion density in the polymer matrix. In particular, when the temperature gradient was applied, electrolyte expansion in the pores increased entropy at the hot side, intensifying ion disorder, and driving ions more actively toward the cold side. In addition, PEG domain insertion within the PVDF‐HFP expanded the free volume upon thermal stimulation, increasing the proportion of ion channels. Furthermore, the thermal expansion reduced polymer crystallinity, which reduced *κ*. Consequently, the onion epidermal cell‐like structure polymer effectively modulated heat transfer and maintained a high temperature gradient, improving the overall TE properties (∼ 0.14 S m^−1^, ∼28 mV K^−1^, and ∼0.041 W m^−1^ K^−1^) (Figure 9c).

In addition, Xing et al. fabricated uniaxially aligned fibrous TD cells via an electrospinning process to reduce heat transfer efficiency while preserving *σ_i_* (Figure 9d) [169]. The uniaxially aligned fibrous structure led to an increase in phonon scattering at the boundaries, which contributed to the low heat transfer efficiency. Furthermore, the uniaxially aligned fibers not only shortened the ion migration pathways but also enhanced the mechanical properties through ion–dipole interactions between PEG and the ionic liquid (EMIM^+^DCA^−^) at the fiber interfaces. As a result, the fibrous TD cells effectively suppressed *κ* (0.18 W m^−^^1^ K^−^^1^) and achieved *σ_i_* and *α* values of up to 0.31 S m^−^^1^ and 11.2 mV K^−^^1^, respectively, along with an enhanced toughness of 53.1 MJ m^−^^3^.

### 6.2 Specific Geometric Architectures for an Amplifying Redox Concentration Gradient in the TG Effect

Structural‐scale engineering strategy also involved maximizing the concentration gradient through hetero‐architectures. By amplifying the concentration gradient of redox pairs, the corresponding cell potentials were enhanced, thereby maximizing energy generation. For example, Sun et al. fabricated a 3D porous conical structure TG cell via ice‐templating and freeze‐drying method (Figure 9e) [170]. The conical TG cell formed a height‐dependent hetero‐structure, which induced distinct surface characteristics at different vertical positions. Under exposure to heat and steam, this hetero‐structure resulted in varying rates of water evaporation along the height of the conical structure, with the apex exhibiting the highest rate (Figure 9f). Convection effect from different water evaporation rates between the bottom and top contributed to intense evaporation at the top, resulting in significantly lower temperature compared to the bottom. Therefore, temperature and concentration gradients were then enhanced, greatly improving the TE performance (Figure 9f,g). Consequently, *α* and *σ_i_* increased from 1.39 mV K^−1^ and 1.63 S m^−1^ to 3.25 mV K^−1^ and 3.06 S m^−1^, respectively, compared to without a water evaporation system.

### 6.3 Stacking Several Cells for Efficient Redox Utilization in the TG Effect

**FIGURE 10:**
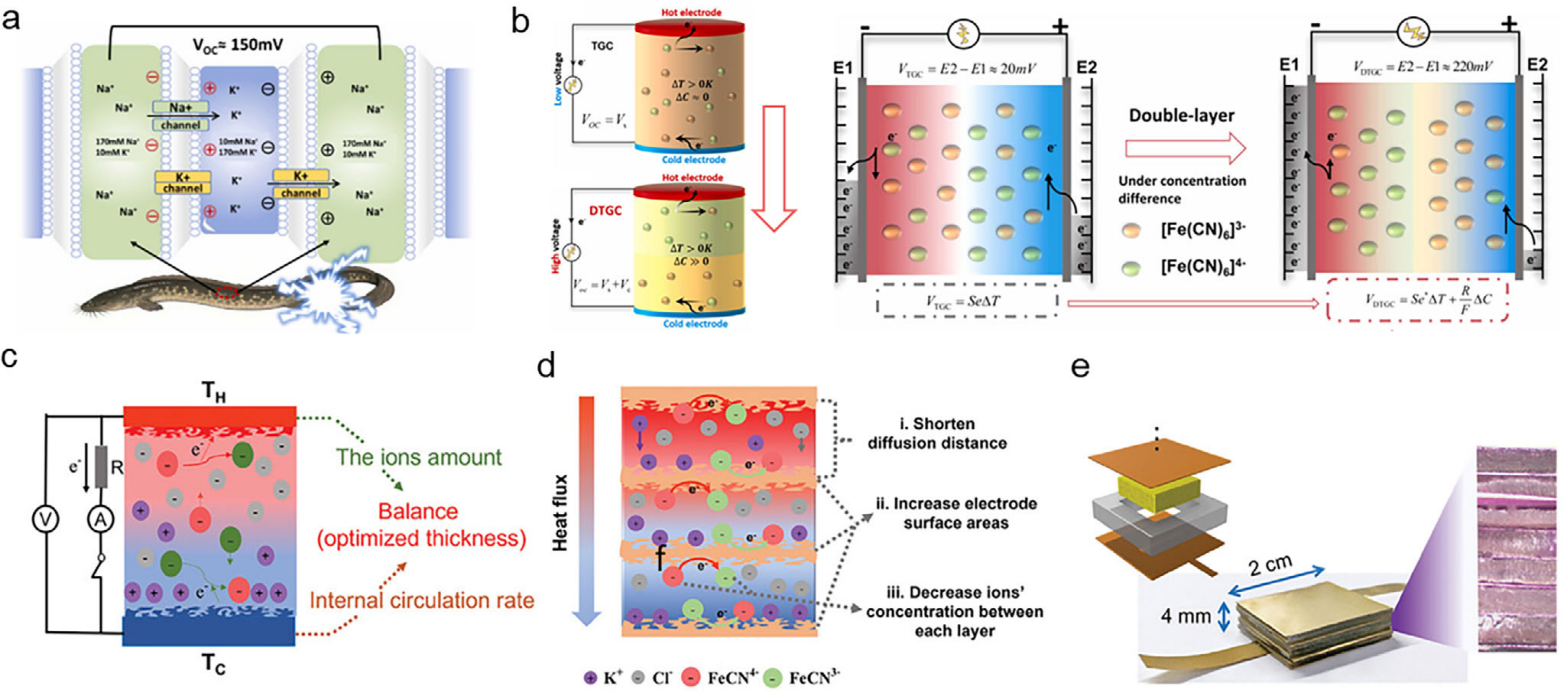
(a) Electrogenesis phenomenon in electric eels. (b) Hetero‐structured TG cells inspired by electric eels. Reproduced with permission [171]. Copyright 2025, Elsevier. (c) The optimization of the thickness based on output performance. (d) Schematic diagram of the multilayer electrode structure and (e) Photographic image of an 8‐layer TG cell. Reproduced with permission [172]. Copyright 2025, Wiley–VCH.

Some researchers have sought to effect structural modification by simply integrating several cells while preserving the original architecture of iTEs [171, 172]. Chi et al. were inspired by electric eels and mimicked their structural features, preserving a large concentration gradient between redox couples in the TG cell (Figure 10a). Specifically, they realized this effect by constructing a hetero double‐layer architecture composed of two layers, each containing only an oxidizing or a reducing species, thereby maximizing the concentration gradient (Figure 10b) [171]. The structure efficiently regulated the migration of redox species at the junction interface, hence restricting convection, which leads to concentration equilibrium across the system. The prominent concentration gradient established by the double‐layer structure across the system maximized the internal potential (∼200 mV), increasing the *α* (2.17 mV K^−1^).

On the contrary, Li et al. regulated the ion migration pathway by stacking TG cells. They systematically investigated the relation between electrode spacing distance and charging time in the TG effect (Figure 10c) [172]. Shortening the distance between the electrodes reduced the ion pathway (a decrease in internal resistance), which decreased the amount of inactive redox species. Consequently, it demonstrated a remarkably fast charging time. In addition, they optimized the TE performance by stacking numerous TG cells with short electrode spacing (Figure 10d). The multi‐stacking structure significantly decreased the charging time from 27 to 8 min, while the power density increased from 5 to 8.1 W m^−2^ as the number of electrode layers increased from 2 to 8.

## 7 Effect of the Polymer Matrix on Mechanical Properties and Self‐Healing

The common role of various functional polymer matrices in QSS‐ionic conductors is to enhance the hardness of materials compared to LS‐ionic conductors [168, 169, 173]. The strengthened hardness expands the application potential of QSS‐ionic conductors. Furthermore, many researchers have recently been pursuing attributes such as exceptional toughness and self‐healing beyond simply improving hardness [173]. This section explores the mechanical characteristics achieved by the polymer matrix.

### 7.1 Stretchability and Mechanical Robustness

**FIGURE 11:**
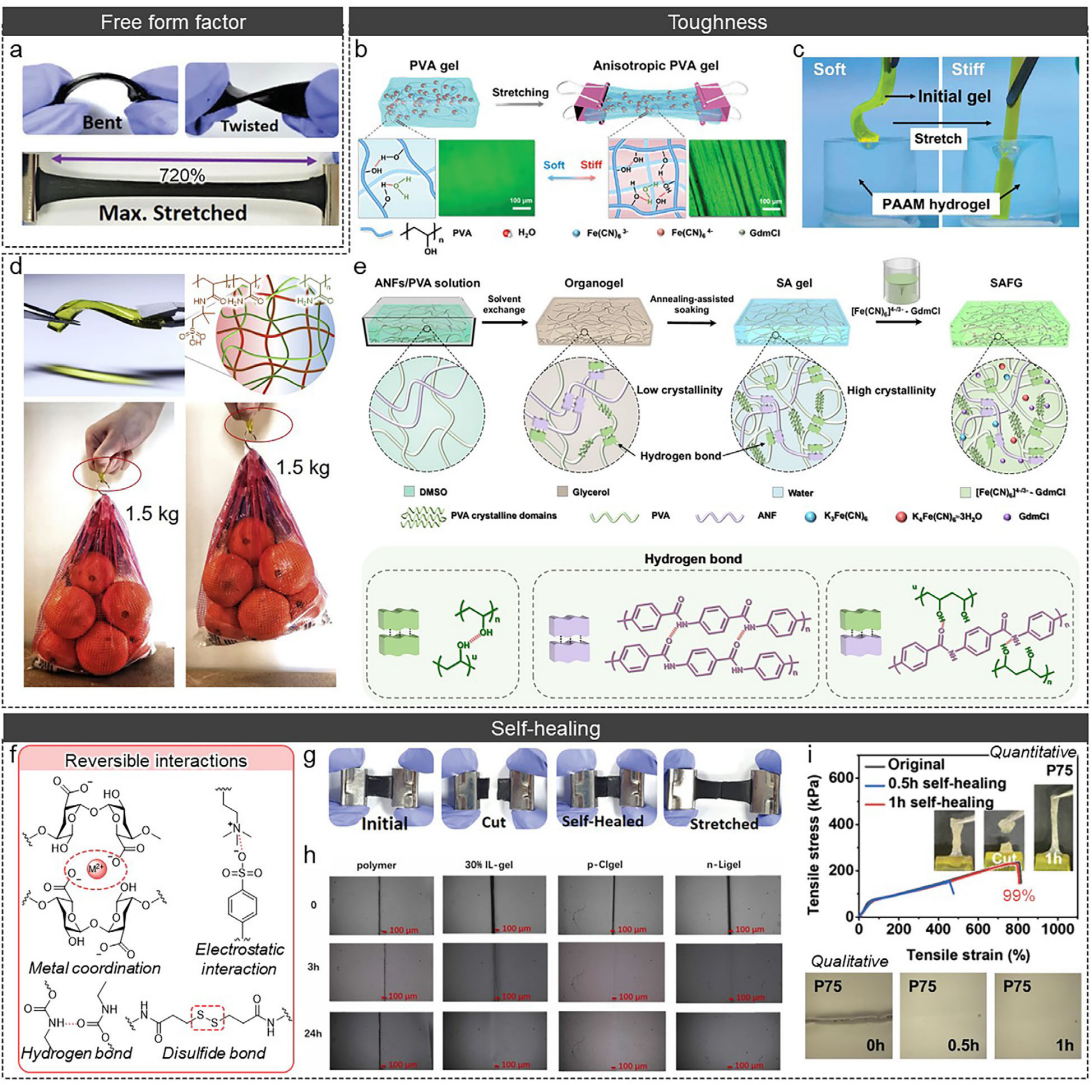
(a) Photographic image of free form factor in QSS‐iTE. Reproduced with permission [11]. Copyright 2021, Wiley‐VCH. (b) Anisotropic polymer via freeze‐thaw and mechanical training, and (c) Photographic image of polymer before and after stretching inserted into PAAm hydrogel. Reproduced with permission [63]. Copyright 2023, Wiley‐VCH. (d) Photographic image of DN polymer and lifting the 1.5 kg bag. Reproduced with permission [64]. Copyright 2021, Elsevier. (e) Illustration of the process of highly dense polymer and multiple hydrogen bonds in the polymer. Reproduced under the terms of the CC‐BY 4.0 license [173]. Copyright 2025, Springer Nature. (f) Various reversible interactions for self‐healing. (g) Photographic image of self‐healing properties. Reproduced with permission [11]. Copyright 2021, Wiley‐VCH. (h) Optical microscopic image depending on the healing time. Reproduced with permission [106]. Copyright 2022, Elsevier. (i) Quantitative and qualitative analysis of self‐healing from the stress‐strain curve and Optical microscopic image. Reproduced with permission [58]. Copyright 2023, Wiley‐VCH.

The polymer matrix provides a 3D network structure, which enhances mechanical strength, while LS‐ionic conductors permeate the polymer matrix. The systems enlarge the free volume of the polymer (lowering the glass transition temperature (*T*_g_) of the polymer). Consequently, polymer chains exhibit vigorous behavior (strain) at room temperature. Moreover, the interconnections (covalent bonding in chemical cross‐linked polymer and entanglement & secondary bonding in physical cross‐linked polymer) between polymers confer restoration to the materials (recovery). The combined effect of strain and recovery is referred to as stretchability, and numerous polymers exhibiting outstanding stretchability and free form factor have been reported in QSS‐ionic conductors (Figure 11a and Table 3) [41, 46, 48, 53, 58, 63, 64, 66, 69, 75, 80, 82, 88, 102, 106, 149, 158, 162, 173, 185].

**TABLE 3:** Comparison of mechanical characteristics (stretchability, toughness, fracture energy, self‐healing) of reported TG and TD cells based on polymers.

Polymer	Electrolyte	iTE type	Stretchability (%)	Toughness (MJ m^−3^)	Fracture energy (KJ m^−2^)	Self‐healing (min, %)	Refs.
P(([EMIM][SPA])‐*co*‐MA)	—	TD	807	118.0	—	60, 99 (R.T)	[58]
P(([APTA][TFSI])‐*co*‐MA)	—	TD	865	133.3	—	60, 99 (R.T)	[58]
P(AAm‐*co*‐AMPS)	Fe(CN)_6_^3‐/4−^/Water	TD	217	—	2.8	—	[64]
PVDF‐HFP	EMITFSI /EMIMCl	TD	1700	—	—	1440, 67 (R.T)	[106]
PVDF‐HFP	EMIMTFSI /LiBF_4_	TD	1700	—	—	1440, 67 (R.T)	[106]
PEO	EMIMDCA	TD	1330	53.1	123.4	—	[162]
Carboxyl‐Functionalized Bacterial Cellulose	EMIMDCA	TD	40	29.7	—	—	[41]
PAA‐PEO	NaCl /Water	TD	1100	7.34	—	—	[46]
PVA‐TOBC	NaClO /Water	TD	1360	22.5	—	—	[48]
PDAC	EMIMTFSI	TD	762	2.9	—	90, almost (60°C)	[102]
PVA	Fe(CN)_6_^3‐/4−^/Water	TG	1300	163.4	—	—	[63]
PVA	Fe(CN)_6_^3‐/4−^/Water	TG	470	—	17.9	—	[158]
PVA‐ANF	Fe(CN)_6_^3‐/4−^/Water	TG	1010	—	368.0	—	[173]
PLAM	HQ/BQ /Water	TG	1703	—	—	20, 99 (R.T)	[149]
PVDF‐HFP/ fluoro‐surfactant	EMIMOtf	TG	505	407.7	—	30, almost (90°C)	[53]
PAAm‐PVA 10%	Fe(CN)_6_^3‐/4^ /Water	TG	133	18.5	—	—	[66]
PNAGA‐F68	Fe(CN)_6_^3‐/4^ /Water	TG	450	—	3.12	—	[69]
PVA‐isotropic	Fe(CN)_6_^3‐/4^ /Water	TG	900	70.7	—	—	[80]
PVA‐stretch	Fe(CN)_6_^3‐/4^ /Water	TG	677	29.1	—	—	[82]
Gelatin‐PAAm	Fe(CN)_6_^3‐/4^ /Water	TG	325	164.1	—	—	[88]
PVA‐stretch	Fe(CN)_6_^3‐/4^ /Water	TG	535	483.2		—	[185]

Stretchable iTEs encounter diverse physical stress, necessitating durable polymer matrices to withstand this. The energy a polymer has to endure before being broken is defined as toughness, and some researchers have attempted to develop polymers with stronger toughness through morphological engineering (Table 3) [63, 75, 158]. For example, Liu et al. adopted a hierarchical structure polymer, produced via a stretching‐induced crystallization method of PVA [63]. Hydrogen bonds are formed between the alcohol groups in PVA through numerous freeze‐thaw cycles. A subsequent stretching procedure leads to an anisotropic crystalline structure (Figure 11b) [63]. This structure dramatically increases the toughness (163.4 MJ m^−3^) while maintaining the stretchable properties of the polymer (Figure 11c).

However, the anisotropic crystalline structures have a specific directionality, and fatigue‐resistant properties also have a directionality, which limits their potential. To address this issue, polymer matrices with a dense and randomly crystalline structure are required [64, 173]. Thus, Lei et al. designed a double‐network structure [64]. The first network served as a robust support matrix, and the second network synergistically enhanced the fracture toughness (2.85 kJ m^−2^) of the system (Figure 11d). Furthermore, Liu et al. created a polymer matrix by incorporating aramide nanofiber (ANF) into PVA [173]. Amide groups in ANF and alcohol groups in PVA formed hydrogen bonds: intramolecular ANFs, intramolecular PVA, and intermolecular ANFs/PVAs. In turn, the polymer chains were rearranged by an annealing process, yielding a highly crystalline structure (Figure 11e). The intermolecular interaction (hydrogen bond) between ANFs and PVAs generates a micro‐separated structure that dissociates and recombines under external stress, enhancing crack resistance (368 kJ m^−2^ fracture energy).

### 7.2 Self‐Healing Polymer Matrices for Mechanical Durability

Unfortunately, if energy surpassing the toughness of polymers is exerted, the stretchable iTEs lose their function. To overcome these challenges, many researchers have investigated self‐healing properties (Table 3) [11, 53, 58, 106]. Self‐healing refers to the phenomenon wherein materials autonomously restore themselves to their original form after damage over time. To achieve self‐healing, a 3D network established via reversible interactions (e.g., metal coordination, hydrogen bond, electrostatic interaction, and disulfide bond (covalent bond)) is required (Figure 11f) [11, 53, 58, 106]. Thus, numerous researchers have designed polymers with functional groups that enable reversible interactions [11, 53, 58, 106]. For example, Malik et al. reported SiO_2_ nanoparticles/polyaniline (PANI): poly(2‐acrylamide‐2‐methyl‐1‐propanesulfonic acid) (PAMPS): phytic acid (PA) organic‐inorganic composite system [11]. The system was composed of hydrogen bond (PANI:PAMPS) and electrostatic interaction (SiO_2_:PANI:PAMPS:PA), showing self‐healing properties (Figure 11g). However, to achieve nearly 100% self‐healing of the composite system, a relative humidity (RH) over 80% was required.

Polymers with high *T*_g_ need high RH or temperature conditions for self‐healing due to restricted chain motions [53]. Thus, self‐healing demands reversible interactions and dynamic polymer chain motions simultaneously. Liu et al. investigated the self‐healing properties of pure PVDF‐HFP, PVDF‐HFP with 30 wt% ionic liquid, and PVDF‐HFP with 30 wt% ionic liquid and ion salt under ambient conditions [106]. Pure PVDF‐HFP exhibited no self‐healing over 24 h, whereas PVDF‐HFP with ions revealed self‐healing properties. Furthermore, the incorporation of an additional ion salt with the ionic liquid resulted in a lower *T*_g_ of the polymer and boosted self‐healing (Figure 11h). In addition, Kim et al. designed polymers including ionic functional groups at the ends of long side chains [58]. The long side chains lowered the *T*_g_ in polymers, facilitating the chain motions, and ionic moieties at the end formed the reversible interactions in the polymer matrix. These properties led to the self‐healing under ambient conditions, which achieved over 99% self‐healing within 1 h (Figure 11i).

### 7.3 Mechanical Properties‐Coupled Ion Transport and Thermoelectric Performance

The polymer matrix simultaneously serves as a mechanical scaffold and as the medium that forms ion transport pathways; hence, mechanical properties inevitably relate to ion migration [67, 69, 174]. For example, polymer networks with high cross‐linking density or reinforced domains usually exhibit improved toughness and fatigue resistance; nevertheless, excessive rigidity hinders polymer segmental motion and impedes ion transport. The restrictions of ion transport result in reduced *σ_i_* and decreased output power [68, 69]. In contrast, highly soft polymer matrices enhance ion transport by enlarging free volume; however, these suffer from mechanical instability and cumulative damage under repeated strain [175]. The trade‐offs underscore the importance of polymer matrix design in achieving a balance between mechanical properties and TE performance.

**FIGURE 12:**
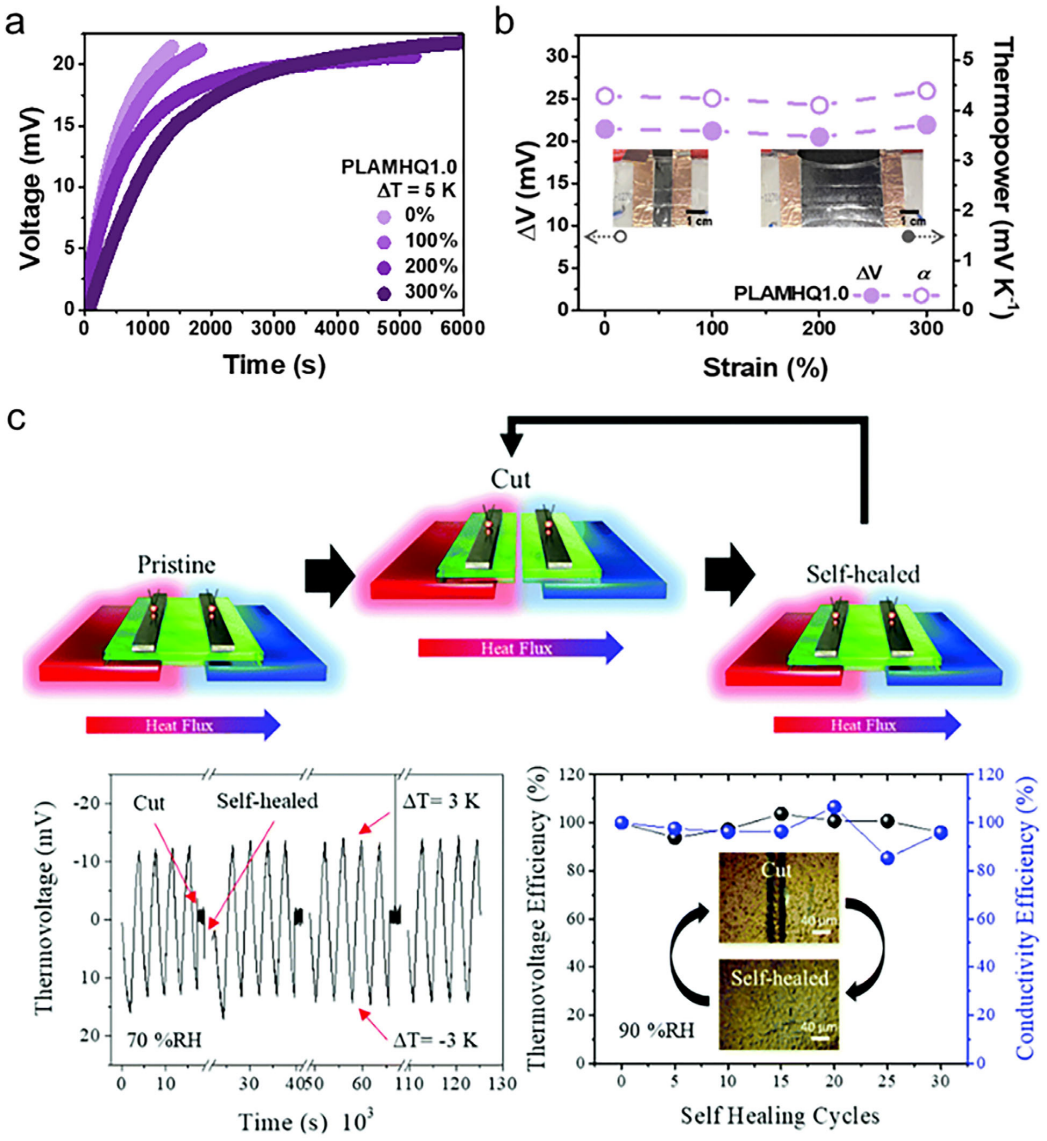
(a) Thermo‐voltage time profile and (b) *α* depending on the various stretching conditions. Reproduced with permission [149]. Copyright 2024, Royal Society of Chemistry. (c) Illustration of the self‐healing process in iTEs, and TE performance after self‐healing. Reproduced with permission [39]. Copyright 2020, Royal Society of Chemistry.

Under practical working conditions, the polymer is inevitably exposed to mechanical deformation, such as bending and stretching. The mechanical deformation leads to a dynamic change of ion transport pathways beyond static materials. For example, uniaxial stretching increases the ion transport pathway and reduces the cross‐sectional area. Therefore, the duration for mobile materials to migrate to the electrode interface is prolonged, requiring a longer period to reach a specific thermo‐voltage (Figure 12a) [149]. While these changes influenced ion conduction by altering ion migration pathways, their impact on *α* has generally been reported to be slight (Figure 12b) [68, 149, 176]. Furthermore, some researchers have confirmed the recovery of *σ_i_* and *α* by self‐healing in cells [39, 53, 58]. The cells restored their initial performance, demonstrating that self‐healing recovers both mechanical and TE properties (Figure 12c). Consequently, the mechanical properties of the polymer primarily affect ion transport and performance stability during deformation, whereas providing limited leverage for *α* modulation through mechanical deformation alone.

## 8 Wearable Ionic Thermoelectric Applications

**FIGURE 13:**
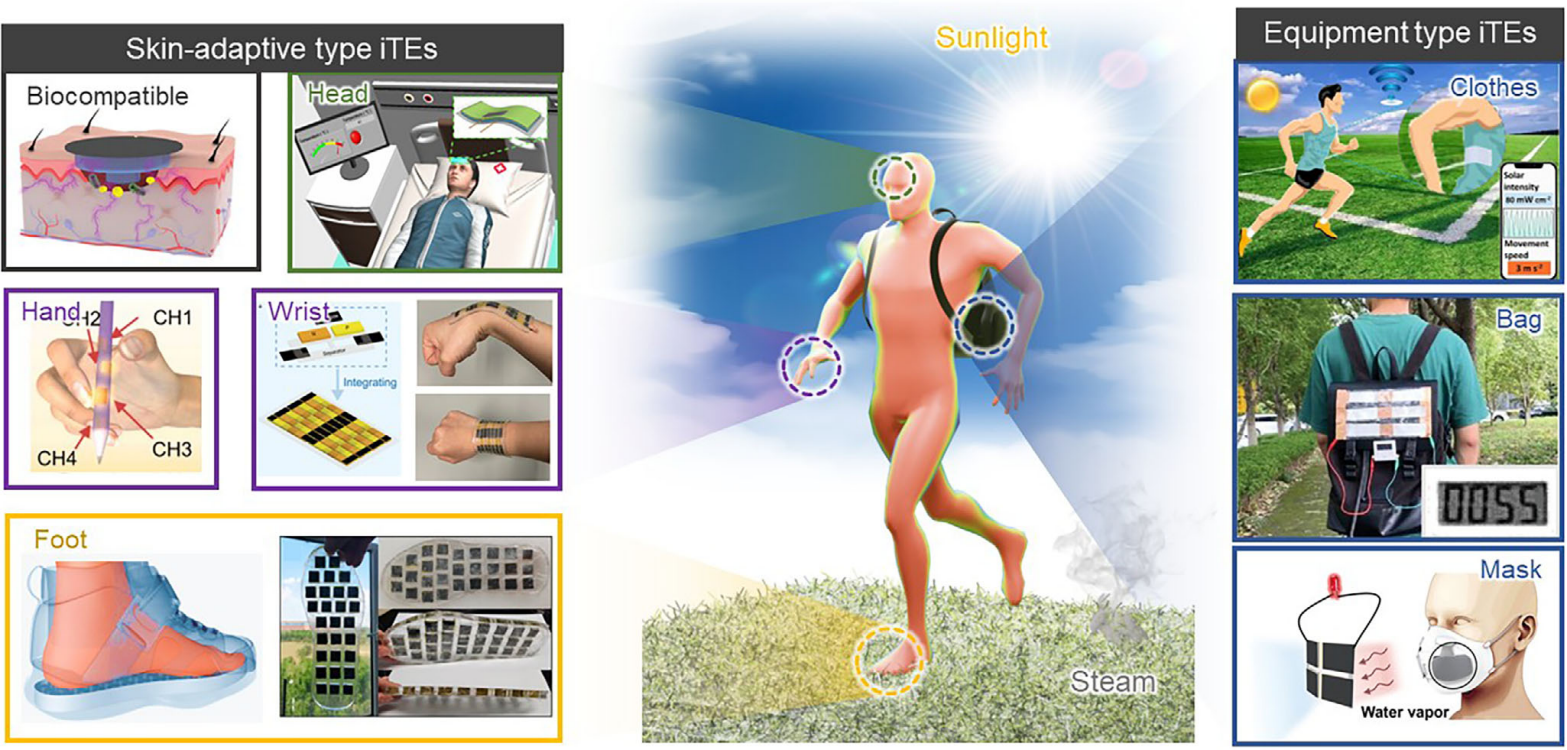
Overall diagram of practical wearable iTE applications. Reproduced with permission [177]. Copyright 2025, Wiley–VCH. Reproduced with permission [178]. Copyright 2021, American Chemical Society. Reproduced with permission [179]. Copyright 2024, Elsevier. Reproduced with permission [76]. Copyright 2022, Wiley–VCH. Reproduced with permission [180]. Copyright 2023, American Chemical Society. Reproduced with permission [67]. Copyright 2023, Elsevier. Reproduced with permission [152]. Copyright 2025, Wiley–VCH. Reproduced with permission [110]. Copyright 2023, Wiley–VCH.

The performance improvements of iTEs, coupled with their outstanding mechanical properties and functionalities such as self‐healing, demonstrated significant potential for next‐generation wearable electronics. With regard to this potential, numerous researchers have developed various wearable devices harnessing the low‐grade heat released by our surroundings (Figure 13) [67, 76, 110, 152, 177, 178, 179, 180]. This chapter provides various wearable applications that employ iTEs and the key factors required in each application field.

### 8.1 Biocompatible Ionic Thermoelectric for Medical Applications

**FIGURE 14:**
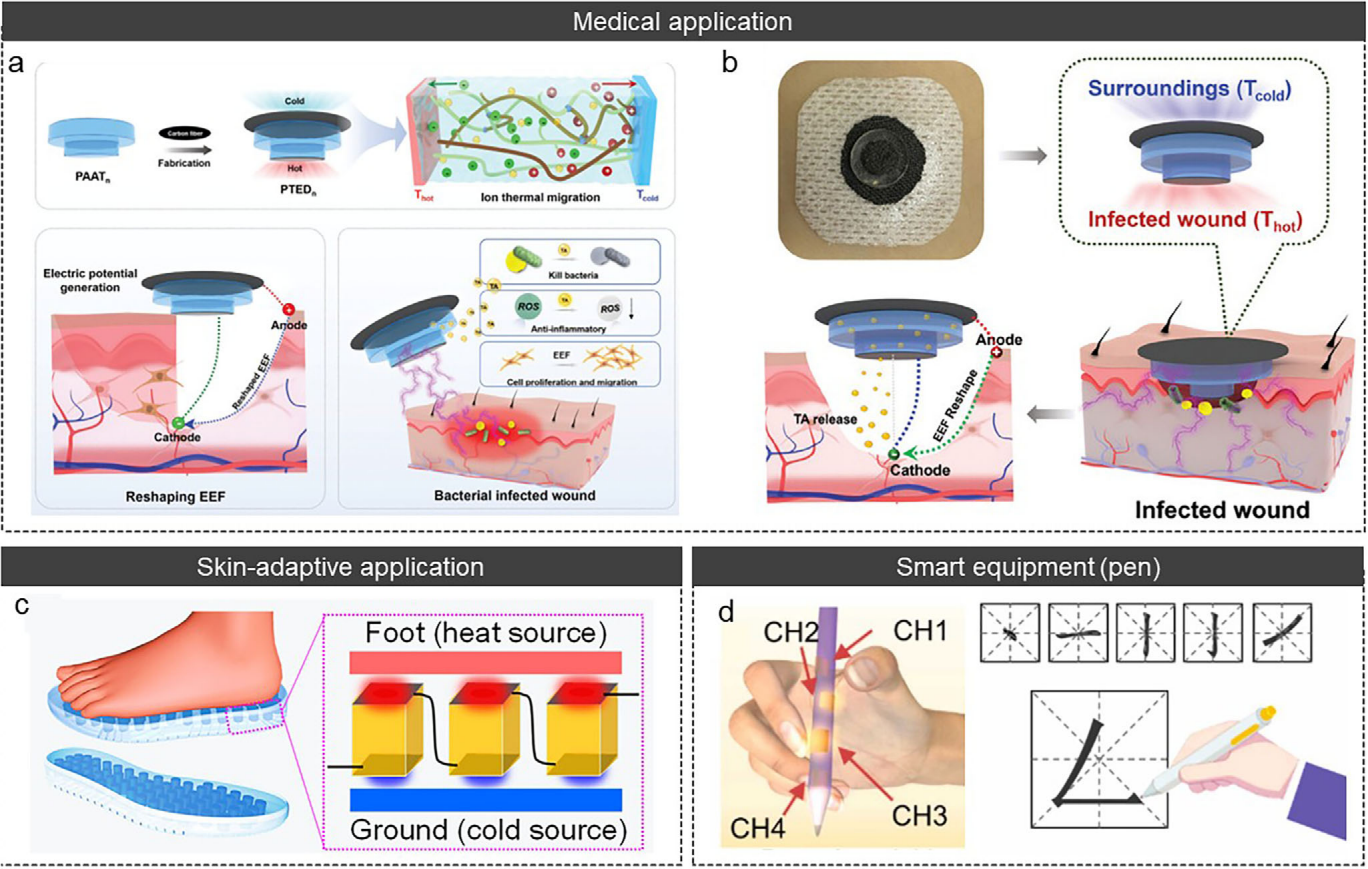
(a) Schematic illustration of biocompatible and wound‐healing TD cells. (b) Photographic image of the biocompatible TD cell and its wound‐healing mechanism. Reproduced with permission [177]. Copyright 2025, Wiley–VCH. (c) Conceptual design of wearable TG cell shoe soles and integrated shoes. Reproduced with permission [180]. Copyright 2023, American Chemical Society. (d) Schematic illustration of a smart pen utilizing finger and hand temperature and a stroke recognition system during handwriting using the smart pen. Reproduced with permission [179]. Copyright 2024, Elsevier.

Polymer gel‐based iTEs generally exhibit higher biocompatibility compared to inorganic materials, making them suitable for next‐generation medical applications when included in healthcare systems. In addition, strong adhesion to skin or biological tissue is crucial for effectively harnessing body heat for medical applications. Considering these requirements, numerous biocompatible iTEs have recently been developed and directly attached to the human body [84, 177]. For example, Gao et al. developed a biocompatible TG cell incorporating a hydrogel with tannic acid, which exerted bactericidal effects in vivo and could be directly attached to the body for energy generation and healthcare, simultaneously (Figure 14a) [177]. Furthermore, tannic acid served as the cross‐linker to form the stable chemically cross‐linked polymer network, and formed the hydrogen bonding with skin tissues. The electrical field generated by the TG cell broadly contributed to tissue regeneration and accelerated wound healing via various processes, including epithelization, nerve repair, and angiogenesis [84, 177]. Consequently, the biocompatible TG cell with tannic acid exhibited robust adhesion (∼1732 J m^−2^) to diverse wound sites on the skin, serving as both a dressing and a monitoring system (Figure 14b).

### 8.2 Practical Skin‐Adaptive Ionic Thermoelectrics on Various Body Parts

The skin‐attachable devices required a platform that adapts to changes in body shape. Therefore, sufficient mechanical strength is necessary to withstand various and continuous deformation (e.g., pressure, strain, and stress from body motion). Furthermore, hydrogel iTEs suffer from performance degradation due to water evaporation during long‐term use. Therefore, stability against the external environment (repeated mechanical deformations and humidity) is required. Therefore, researchers have focused on designing device architectures compatible with various body motions for stable working [177, 178, 179, 180]. Specifically, skin‐attachable iTEs have been reported for the wrist, forearm, arm, and forehead, where the temperature gradient is most pronounced, to maximize the temperature gradient with the surroundings (Figure 14c) [180]. Furthermore, Zhang et al. applied TG to the soles of the feet, where a significant temperature gradient is formed during physical activity [180]. Walking caused substantial friction with the ground, yielding high thermal energy in the soles, leading to a thermo‐voltage of 1.2 V. Notably, the iTE showed approximately a 5% decrease in *α* after 500 stretching (100%). Meanwhile, a slight drop in *α* was observed after 7 days, demonstrating significant resistance to mechanical deformation and environmental exposure. The results demonstrated that iTEs effectively harvest energy when strategically positioned on various body parts, rendering them promising power generators for a wide range of wearable electronics.

Moreover, we contact several objects in our daily lives, which causes heat dissipation within those objects. Thus, it provided opportunities for harnessing body heat during daily activities [179]. Shang et al. integrated TG with a smart pen, which autonomously worked from the finger temperature during handwriting (Figure 14d) [179]. The TG exhibited consistent thermo‐voltage under repeated stress and bending conditions, demonstrating mechanically stable working under repeated deformation. Moreover, the addition of dimethyl sulfoxide as a solvent medium in iTE preserved approximately 90% of the initial weight for 30 days, indicating that the internal composition of iTE was almost constant. Consequently, the working of the smart pen was successfully verified via finger heat, confirming that it monitors the letters during handwriting.

### 8.3 Photothermal‐Driven Wearable Ionic Thermoelectrics

**FIGURE 15:**
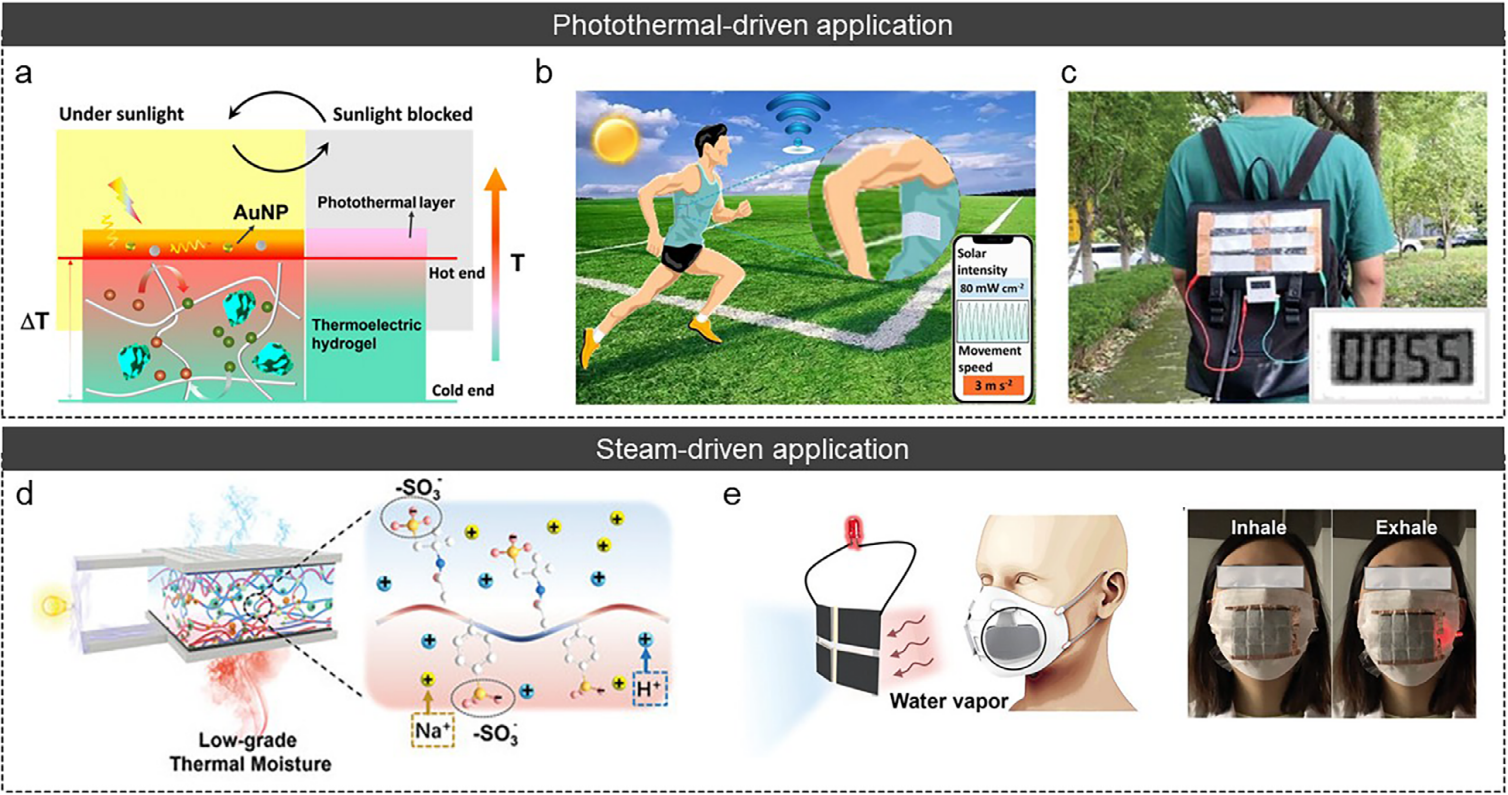
(a) Schematic of photothermal‐driven TG cells. (b) patch‐type TG cell on clothing. Reproduced with permission [67]. Copyright 2023, Elsevier. (c) Photothermal‐driven TG cells integrated on a bag. Reproduced with permission [152]. Copyright 2025, Wiley–VCH. (d) Illustration of steam‐driven TD cells and (e) steam‐driven TD cells using the hot moisture from human breath. Reproduced with permission [110]. Copyright 2023, Wiley–VCH.

Photothermal energy is a ubiquitous factor in our environment, and certain researchers have manufactured iTEs that exploit it. For example, Yang et al. devised iTEs using solar heating or radiative cooling [67, 181]. In particular, a film composed of the photothermal materials (Au nanoparticles) was coated on the surface of the iTEs to further increase the temperature gradient (Figure 15a). To facilitate effective sunlight exposure, the iTEs were attached to clothing patches (Figure 15b) and bags (Figure 15c), thereby maximizing energy generation.

### 8.4 Steam‐Driven Wearable Ionic Thermoelectrics

Since thermal energy in the surroundings is often accompanied by ambient moisture, a composite steam has been considered as an efficient energy source. Steam, absorbed in a specific direction, formed a substantial concentration gradient due to ion dissociation along with the temperature gradient, which enhanced the TE performance compared to the temperature gradient by itself [110, 170]. Furthermore, a large surface area of the TE device was crucial for increasing the amount of absorbed water, thereby improving TE performance. However, excessive water uptake induces structural collapse or deformation of the polymer network. Therefore, a mechanically robust polymer network is required to maintain TE performance. For instance, Yang et al. designed a porous‐structured iTE device powered by heated steam, enhancing the TE performance by amplifying the concentration gradient (Figure 15d) [110]. Under continuous steam exposure (approximately 118 h), the porous structure exhibited continuous working without any noticeable structural deformation, while simultaneously achieving maximized TE performance. Moreover, they developed a wearable TD cell that harvests energy from human breath (Figure 15e).

## 9 Conclusions and Perspectives

iTEs have garnered considerable interest in recent decades, demonstrating substantial progress in both fundamental understanding and practical performance. In particular, QSS‐iTEs exhibited unique properties compared to LS‐iTEs, owing to the incorporation of polymer matrices. Functional polymers not only govern ionic transport but also regulate mechanical robustness and device adaptability, making polymer design a central strategy for advancing iTE technologies. Therefore, this review has highlighted the role of polymers across molecular‐, micro‐, and macro‐scale engineering, as well as their influence on TE output, mechanical properties, and multifunctionality. Furthermore, recent demonstrations of wearable and environmental energy‐harvesting applications highlight the growing potential of iTEs in our surroundings.

Despite these advances, significant challenges remain. For TD cells, the high *α* is offset by limited practicality due to their EDLC‐like working mechanism, which emphasizes energy storage rather than continuous energy generation. In contrast, TG cells offer a pathway to overcome these limitations, and recent developments have markedly improved their performance [56, 62, 117]. However, their performance indicators remain below commercialization standards.

In particular, the figure of merit (*ZT*) calculated by the *α*, *σ_i_*, and *κ* (in Equation 16) has rarely surpassed one, the threshold commonly considered necessary for competitive TE technologies [178, 182].

(16) $$ZT=\frac{\sigma_{i}\alpha^{2}}{\kappa T}\;$$

Moreover, there are other TE performance parameters, thermal energy conversion (*η*) and Carnot relative efficiency (*η_r_*) as follows: Equations (17) and (18):

(17) $$\eta =\frac{P_{max}}{P_{heat}}\;$$

(18) $$\eta_{r}=\frac{\eta }{\eta_{c}}=\frac{\sqrt{1+ZT_{avg}}-1}{\sqrt{1+ZT_{avg}+T_{c}/T_{h}}}\;$$

where *P_max_*, *P_heat_*, and *η_C_* denote the maximum output power density, heat input power density, and Carnot efficiency, respectively. *η_r_* is a quantitative indicator of the conversion of thermal energy into electrical energy and is considered commercially viable when it exceeds 5% [80, 104, 167, 185]. Although there have been instances of *η_r_* reaching up to 3% among reported QSS‐TG cells [63], further developments are still required to achieve commercialization.

From a practical perspective, iTEs are primarily evaluated based on their output power and *η_r_*, as these factors significantly affect their usefulness in practical applications [80, 104, 167, 185, 186, 187]. The required output power for operation varies with the intended applications, rendering power generation capacity a key design consideration [186, 187]. Since output power is determined by both voltage and current, serial integration of numerous iTE units provides a straightforward approach to raise the total thermo‐voltage, thereby enabling higher power generation when current is not severely constrained.

However, this increase of voltage and power does not inherently improve the efficiency of heat‐to‐electrical conversion. The *η_r_* is determined by the fundamental properties of each individual iTE unit. A *η_r_* of approximately 5% is frequently suggested as a minimum requirement for commercialization [80, 104, 167, 185]; nevertheless, higher levels are clearly desirable for the effective utilization of low‐grade thermal energy. Following Equation (18), achieving high *η_r_* demands a substantial *ZT*, arising from the complementary contributions of high *α*, high *σ_i_*, and low *κ*. Therefore, further advancements in iTEs rely on design strategies that simultaneously optimize these mutually dependent parameters, facilitating both effective energy conversion and practical output power.

### 9.1 Molecular‐Scale Engineering: Expansion of the Working Temperature Range

A higher temperature gradient in the iTE system results in a higher output voltage. Thus, QSS‐ionic conductors exhibiting extraordinary thermal stability are required [184]. However, reversible interactions by functional groups in polymers are easily broken as the temperature rises, eventually leading to the collapse of the 3D structure. The unstable 3D structure restricts the migration path of mobile ions and redox couples, degrading the TE performance. Thus, iTEs exhibited a limited working temperature range [184]. To improve the working temperature range of iTE by forming a stable 3D polymer matrix, Li et al. added a cross‐linker (glutaraldehyde (GTA)) into a gelatin matrix for a chemically cross‐linked polymer matrix [153]. The chemically cross‐linked gelatin achieved stable *σ_i_* by preserving the ion pathway even at high temperature.

Moreover, besides the collapse of the 3D structure, water evaporation in the polymer matrix remains a challenge that constrains the working temperature range of iTEs [185]. In the polymer matrix, water is present in three states (i.e., free water, immobilized water, and bound water) depending on the interaction between the polymer and water, with free water being the most easily evaporated [188]. Furthermore, immobilized and bound water possesses a freezing point below 0°C [143]. Consequently, increasing the proportion of immobilized and bound water by regulating polymer–water interactions can broaden the working temperature range (below 0°C and above 100°C). The extended working conditions facilitate operation across a wide temperature range, covering high and low temperatures, and additionally inhibit water evaporation between 60°C and 70°C, ensuring stable device working.

### 9.2 Molecular‐and Micro‐Scale Engineering: Long‐Term Cycle Stability

Ion salts and functional groups that form hydrogen bonds and electrostatic interactions associate with the surrounding water molecules, indicating substantial hygroscopicity. The hygroscopicity causes fluctuations in the water content within the polymer matrix depending on the external RH conditions [96, 189]. Furthermore, it is difficult to maintain consistent RH conditions. Consequently, the water retention in the polymer matrix easily changes depending on the surroundings, which affects the repeatability and long‐term stability of iTEs. In addition, the *κ* of water is 0.6 W m^−1^ K^−1^, which is relatively high compared to those of the polymers (approximately 0.1 W m^−1^ K^−1^) [190]. To achieve long‐term cycle stability and high TE performance (*ZT* and *η_r_*), it is crucial to develop QSS‐ionic conductors with minimal water dependence. QSS‐ionic conductors with low hygroscopicity usually involve bulky ionic liquids having high molecular weight for broad delocalization of electron density [191]. However, their high molecular weight typically results in slow ion dynamics (under 1 S m^−1^), leading to a low current and output power. Thus, to simultaneously attain cycle stability and TE performance (*σ_i_* and current), polymer matrices with elaborate ion channel structures are required.

### 9.3 Micro‐Scale Engineering: Exploration of Novel Redox Couples and The Formation of Environments for the Reaction

Prevalent redox couples (e.g., Fe(CN)_6_^3−/4−^, Co(bpy)_3_^2+/3+^, Fe^2+/3+^, and I^−^/I_3_^−^) exhibit fixed entropy differences, which exhibit a *α* of approximately 1–1.5 mV K^−1^ [162, 163, 164]. Therefore, some researchers have sought to discover new redox couples [149, 192, 193, 194]. Guo et al. proposed a novel strategy that utilized alternative redox couples instead of relying on temperature‐dependent redox reactions [192]. They used benzoquinone (BQ)/hydroquinone (HQ) for a net TG effect, which generated a voltage (6.7 mV K^−1^ of *α*) depending on the pH of the surroundings.

Kim et al. designed an anionic polymer with a self‐regulating pH capability for an n‐type QSS‐TG cell based on HQ [147]. The self‐regulating pH led to a higher HQ concentration than the BQ concentration. Moreover, in a typical polymer matrix, H^+^ migrates rapidly via the Grotthuss mechanism [193]; however, the anionic moieties in the polymer interact with H^+^, hindering its transport and thereby modifying the thermodynamic equilibrium. Consequently, tailoring the thermodynamic equilibrium via self‐regulating pH and selective transport facilitates the effective oxidation reaction (−4.29 mV K^−1^ of *α*) on the cold side. Thus, innovative redox reactions and their associated environments possess significant promise for enhancing performance.

### 9.4 Macro‐Scale Engineering: Suppressing the Thermal Diffusion by the Phonon Effect

The development of a temperature gradient within a system prompts thermal diffusion, which decreases the temperature difference between the hot and cold sides. As a result, the temperature gradient collapses over time, restricting the thermophoresis of mobile materials. Consequently, controlling thermal diffusion is crucial for achieving a stable and large temperature gradient within a system [168, 169]. However, modulating *κ* is challenging through molecular and morphological engineering. Therefore, several researchers have taken a structural engineering approach, exploring various porous structures [168, 195]. Such frameworks effectively confined heat and suppressed thermal diffusion, thereby maintaining the temperature gradient within the systems [168]. However, structural engineering inevitably hinders not only thermal diffusion but also ion migration, because heat conduction couples with ion diffusion, presenting a critical drawback [196]. While thermal diffusion through phonon conduction can be regulated by controlling the interface between the polymer matrix and the electrolyte, thermal diffusion via ion diffusion remains unrestrained [168]. Consequently, the designs of novel architectures and electrolytes are required that selectively suppress thermal diffusion while facilitating rapid ion migration.

Consequently, these future directions highlight quantitative performance for QSS‐iTEs, therefore outlining a realistic pathway to commercialization. Further advancement necessitates *α* on the order of the highest reported values, *σ_i_* approaching those of LS‐iTEs, and long‐term working stability across wide temperature ranges.

Beyond scientific performance, other practical challenges must be overcome for the commercialization of QSS‐iTEs. For example, reproducible and scalable fabrication, such as casting and printing processes, is crucial for achieving a consistent large‐scale device setup. Moreover, durable encapsulation, including elastomeric coating with low *κ* and high moisture resistance, is essential for long‐term stability. In addition, seamless integration requires reliable ion‐electron transduction layers and mechanically robust electrode interfaces. Finally, cost‐related considerations are essential factors for system‐level applications.

**FIGURE 16:**
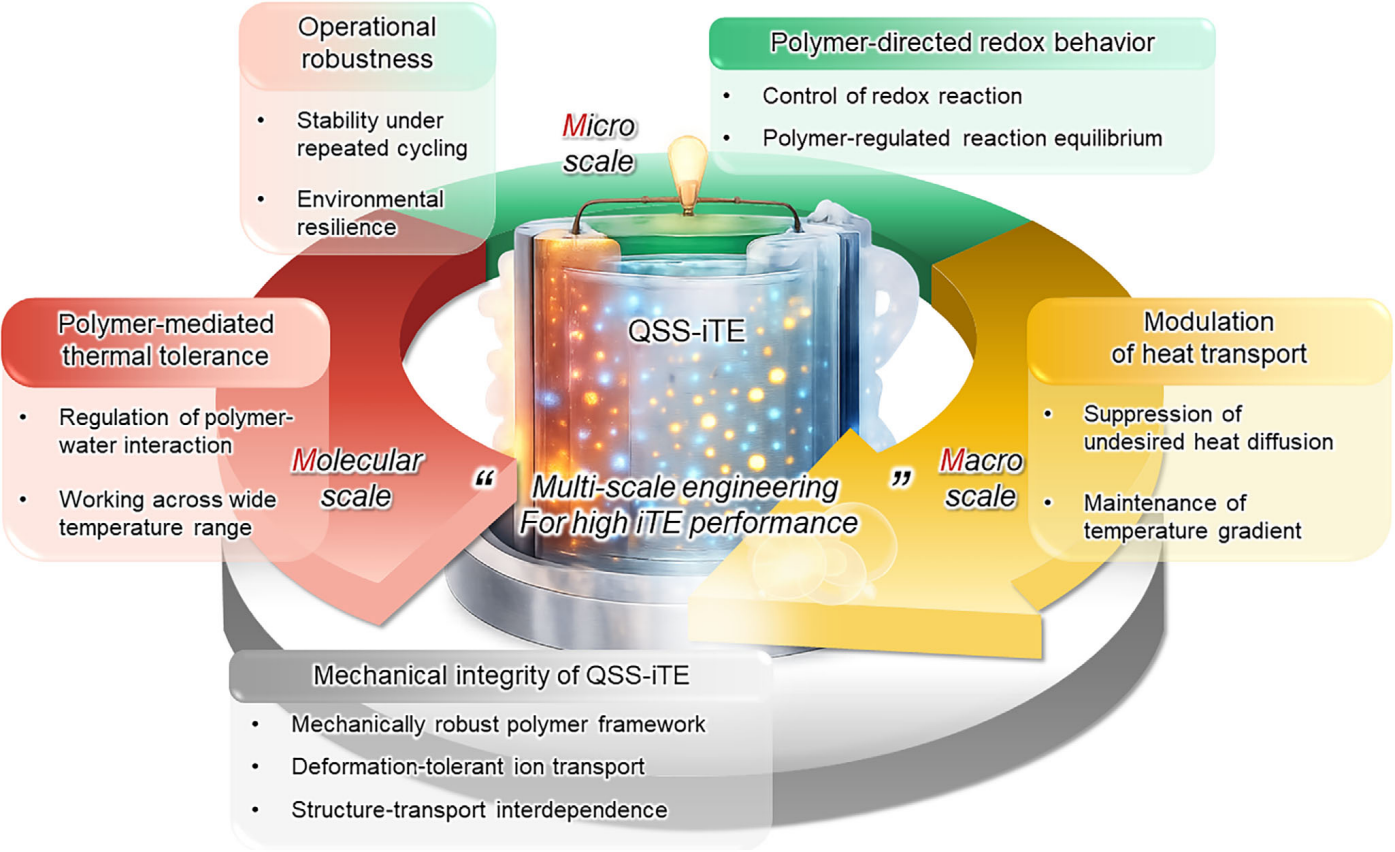
Overall perspective of iTEs from molecular‐, micro‐, and macro‐scales.

In summary, several functional polymer matrices contributed to advancing QSS‐iTEs by simultaneously enhancing TE output and mechanical robustness through tailored 3D structures and polymer‐ion interactions (Figure 16). Furthermore, future progress in multifunctional polymer design, micro‐scale engineering with novel redox couples, and macro‐scale architectures that suppress thermal diffusion via the phonon effect is expected to further boost ZT and *η_r_*, paving the way for the practical realization of iTE‐based energy harvesting devices. Overall, these innovations position TG cells as highly promising energy generators for next‐generation wearable electronics and energy‐harvesting platforms.

## Conflicts of Interest

The authors declare no conflicts of interest.

## Data Availability

No primary research results, software, or code have been included, and no new data were generated or analyzed as part of this review.
